# Whole-mantle convection with tectonic plates preserves long-term global patterns of upper mantle geochemistry

**DOI:** 10.1038/s41598-017-01816-y

**Published:** 2017-05-12

**Authors:** T. L. Barry, J. H. Davies, M. Wolstencroft, I. L. Millar, Z. Zhao, P. Jian, I. Safonova, M. Price

**Affiliations:** 1 Department of Geology, University of Leicester, Leicester, LE1 7RH UK; 2 School of Earth and Ocean Sciences, Cardiff University, Cardiff, CF10 3AT Wales UK; 3 JBA Risk Management, Broughton Hall, Skipton, North Yorkshire BD23 3AE UK; 4 NERC Isotope Geosciences Laboratory, British Geological Survey, Keyworth, Nottingham, NG12 5GG UK; 5 School of Earth Science and Resources, China University of Geosciences, Beijing, 100083 China; 6 Beijing SHRIMP Centre, Institute of Geology, Chinese Academy of Geological Sciences, Beijing, 100037 China; 7 Sobolev Institute of Geology and Mineralogy SB RAS, Novosibirsk, 630090 Russia; 8 Novosibirsk State University, Novosibirsk, 630090 Russia

## Abstract

The evolution of the planetary interior during plate tectonics is controlled by slow convection within the mantle. Global-scale geochemical differences across the upper mantle are known, but how they are preserved during convection has not been adequately explained. We demonstrate that the geographic patterns of chemical variations around the Earth’s mantle endure as a direct result of whole-mantle convection within largely isolated cells defined by subducting plates. New 3D spherical numerical models embedded with the latest geological paleo-tectonic reconstructions and ground-truthed with new Hf-Nd isotope data, suggest that uppermost mantle at one location (e.g. under Indian Ocean) circulates down to the core-mantle boundary (CMB), but returns within ≥100 Myrs via large-scale convection to its approximate starting location. Modelled tracers pool at the CMB but do not disperse ubiquitously around it. Similarly, mantle beneath the Pacific does not spread to surrounding regions of the planet. The models fit global patterns of isotope data and may explain features such as the DUPAL anomaly and long-standing differences between Indian and Pacific Ocean crust. Indeed, the geochemical data suggests this mode of convection could have influenced the evolution of mantle composition since 550 Ma and potentially since the onset of plate tectonics.

## Introduction

Many of Earth’s unique planetary characteristics are the result of plate tectonics. Over time, rocks formed at the surface mix back into the mantle below, and thus modify the chemistry of the Earth’s interior (e.g. refs 1, 2). The upper and lower mantle have contrasting viscosities^3^, so that the upper mantle is generally regarded as a well-mixed reservoir (e.g. ref. 4) depleted of elements that contribute to crustal formation (the crust being enriched in these elements), whereas the lower mantle is thought to convect far more sluggishly, preserving heterogeneity: recycled materials delivered to the lower mantle evolve very slowly, as inferred from distinct geochemical signatures from the radiogenic ingrowth of radio-nuclides (e.g. ref. 1). This view of a viscously two-layer mantle has been the focus of modelling and explanations for regional geochemical variations in oceanic crust, which is formed of mid ocean-ridge basalts (MORB) by partial melting in the upper mantle^5^. In this view, deviations from a standard upper mantle melt composition (termed ‘Normal-MORB’) are typically explained by influxes from buoyant lower mantle plumes or incorporation of shallow, lithospheric material^5^.

Significant differences in isotope chemistry between Indian Ocean MORB and Pacific MORB were first described in the late 1980’s with Sr and Pb isotopes^6^; along with MORB crust (depleted mantle source), these studies including ocean island basalts (from enriched mantle sources) defined the so-called DUPAL^6^ and SOPITA^7^ anomalies. Many studies have focussed on the chemical origins of these anomalies (e.g. ref. 8), generally thought to reflect recycling of parts of the lithosphere into the mantle. Consensus on a unified model has remained elusive due to too many unquantifiable variables such as amount of material recycled, which part of the lithosphere recycled^9, 10^, and the timing of incorporation into the convecting mantle^11, 12^. Suggested recycled components have included lower continental crust^11^, sub-continental lithospheric mantle^10, 11, 13^, altered ocean crust ± pelagic sediments^6^, and subduction modified mantle^9^, as well as material from large low shear velocity provinces^14, 15^. As no consistent model can account for all lines of isotopic evidence, agreement also cannot be reached on whether the anomalies are perpetually produced^16^, are generated during supercontinent break-up^10, 13^ or have existed since the start of plate tectonics^8^.

Focusing on the depleted mantle-MORB evidence for isotopic differences between Indian- and Pacific Ocean MORB, the Australian-Antarctic Discordance is clear evidence of a sharp transition between the two, leading to suggestions of a mantle convection boundary^17^, potentially separated by remnant slab material^18^. Importantly, for understanding mantle convection, isotopically-different ocean crust indicates: (1) the existence of at least two geographically separate, depleted upper mantle compositions, and (2) given that radiogenic isotopes accumulate very slowly, the two mantle compositions must have remained unmixed for 100’s millions of years. Moreover, there is increasing evidence that the distinctive compositions within the upper mantle have persisted for 100’s millions of years, despite plate re-organisations. For example, Indian Ocean MORB composition has persisted since at least upper Palaeozoic times, long before the Indian Ocean formed^13, 19, 20^. This evidence raises fundamental questions about how heterogeneities might be *preserved* within the upper mantle and counters any model of ubiquitous mixing throughout the upper mantle, suggesting that differences with height through the mantle (radial layering) are not the only form of long-duration mantle segregation.

This paper sets out to examine how long-term segregation can persist within the mantle, particularly regarding the maintenance of discrete large-scale reservoirs within the upper mantle. Precise paths taken by packets of circulating mantle material are understandably difficult to constrain because of the mantle’s inaccessibility, the long-time-scales involved and the complexity of heterogeneities on all scales^21, 22^. Hitherto, the best constraints derive from analysis of particle tracing in mantle circulation models that include present-day plate boundaries (e.g. ref. 22). We undertake a step-change with new models that combine (1) spherical simulations using the 3D finite element code TERRA^23, 24^ with (2) reconstructions of Earth’s plate configurations through geological history^25, 26^ to model mantle circulation over 100’s millions of years, with (3) the use of passive tracer particles to explore how distinct regions of the upper mantle are able to remain isolated from one another over 100’s millions of years. TERRA solves equations for velocity, temperature and pressure, conserving mass, momentum, and energy^27^ with an Earth-like convective vigour (see Methods). Significantly, (4) we validate the models using long-lived radiogenic isotopes sampled from known MORB ophiolites across Europe and Asia.

**Figure 1:**
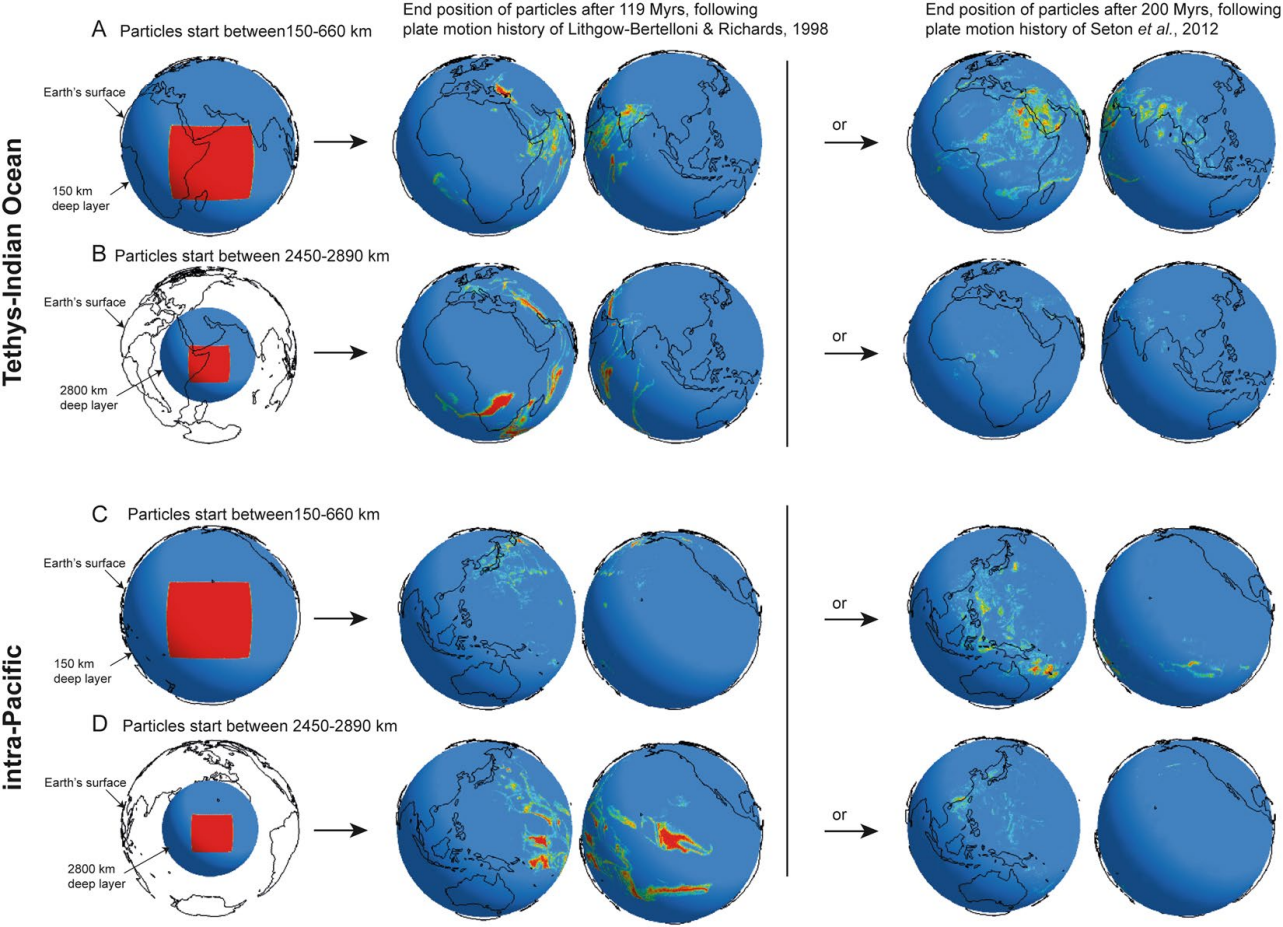
Starting positions and end results for particles tracked for two different plate motion histories beginning at two starting depths. (**A** and **B**) Particles started within a *Tethys-Indian Ocean* geographic location at depths between 150–660 km (**A**) and 2450–2890 km (**B**). The results show the end position of the passive particles following the 119 Myr history of Lithgow-Bertelloni & Richards^25^ and after 200 Myrs following the plate motion of Seton *et al*.^26^. (**C** and **D**) Show the same model conditions, but for particles started in an intra-Pacific geographic locality. Particles started in both upper and lower mantle beneath the Tethys-Indian Ocean circulate to the S. Atlantic but not the Pacific, in both plate motion histories. Those particles started beneath the Pacific spread towards Pacific subduction zones, but do not spread laterally to the Indian Ocean. Images were generated using visualisation software MantleVis; MantleVis is an in-house, unlicensed, distributed visualization tool written in C++ using the OpenGL API, and makes use of the OpenGL DisplayList tool (http://pcwww.liv.ac.uk/~aeh/Software/MantleVis.htm & http://www.helix.cf.ac.uk/helix/?page_id=4). Projections were selected to show all particles present on each layer.

To simulate plate motion history we use surface velocity boundary conditions^27^ from the well-established plate configurations of Lithgow-Bertelloni and Richards^25^ (**LBR** hereafter) spanning 119 Ma, and then using more up-to-date reconstructions by Seton *et al*. of plate motion history going back to 200 Ma^26^ (**Seton** hereafter). Our models using these two different plate history reconstructions, each explore how different regions of the mantle might have circulated as a result of plate motion history (no density or chemical parameters are applied in the models for different mantle lithologies, therefore only spatial distribution assessed). In order to trace the convection pathways, passive marker particles are introduced at three depth ranges: ‘upper mantle’ 150–660 km; ‘mid-mantle’ 800–1200 km; and ‘lower mantle’ 2450–2890 km, located beneath two geographically-defined regions: *Tethys-Indian Ocean* (20°N–20°S, 25°E–70°E) and *intra-Pacific* (20°N–20°S, 135°W–180°W) (Fig. 1; Methods and Supplementary Information Part 1). A 30x viscosity contrast between the upper and lower mantle was used but, otherwise, material was allowed to convect freely in response to plate motions. The results are used to examine material transfer from the upper mantle to the lower mantle and also its return. In particular we consider the behaviour of lateral convective flow within the mantle, as this affects how large-scale chemical heterogeneities might have persisted within the upper mantle during convective circulation.

**Figure 2:**
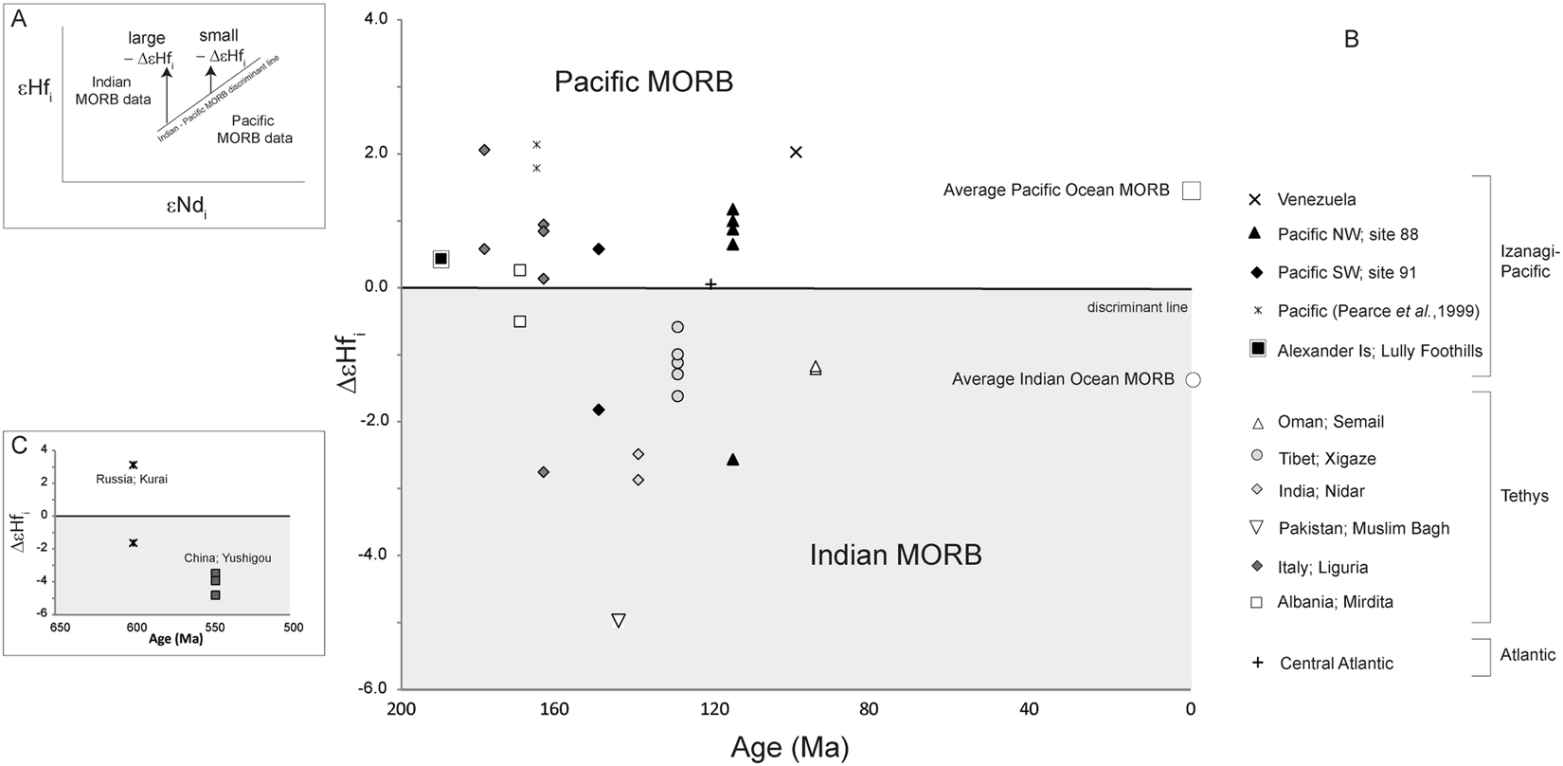
Age of ophiolite basalts (symbols in key) versus ΔεHf_i_ (explanation of notation in top inset). (Lu-Hf decay constant = 1.867 × 10^−11^ ^55^, CHUR values of ^176^Lu/^177^Hf = 0.0336 and ^176^Hf/^177^Hf = 0.282785^56^. Indian–Pacific MORB discriminant line and calculation parameters from Pearce *et al*.^29^; ΔεHf_i_ = 1.6 εNd_i_ – εHf_i_. [Note: Pearce *et al.* ^29^, referred to parameter as ΔεHf_P/I_]. Negative ΔεHf_i_ values indicate sample affinity with Indian Ocean MORB chemistry. Such compositions are evident in the signatures of Tethyan basalts, even in 550 Ma MORB basalts from early Paleo-Tethys (bottom inset; China: Yushigou and Russia: Kurai). See Supplementary Information Part 2 for sample details. Average values for present-day Indian and Pacific MORB calculated from PetDB database and references therein.

Finally, to validate the significance and longevity of discrete convective regimes, that appear to suggest minimal interaction, we analysed Hf-Nd isotopes for depleted upper mantle compositions (MORBs) that span the duration of the model runs. Long-duration heterogeneities in the upper mantle have been proposed from Pb-isotope evidence that Indian Ocean MORB have similar isotope characteristics to MORB from Tethys Ocean remnants; in the Mesozoic and Upper Palaeozoic, Tethys occupied a region similar to that of the present Indian Ocean^13, 20^. Some doubt on the significance of the original works comes from evidence that Pb is highly mobile in the marine and subduction environment^28^. Here, we have analysed Hf-Nd isotopes that are considered to better resist modification during low-temperature sea floor alteration and fluid mobility during subduction, and therefore preserve the composition of the basalt at the time of its formation. Discrimination of present-day Indian Ocean MORB from Pacific Ocean MORB, using Hf and Nd isotopes, has been used successfully to determine mantle provenance in the western Pacific^29, 30^, easternmost Indian Ocean^9^, and in the SW Indian Ocean^31^. We analysed low-grade metamorphosed MORBs from (1) six ophiolites across Europe and Asia (18 samples), (2) two ophiolites and three oceanic sites of old Pacific basalts to assess Pacific mantle compositions (12 samples), and (3) one example of old Atlantic MORB that formed at the margins of the Tethyan-Indian Ocean (2 samples; Fig. 2; Methods and Supplementary Information Part 2 for further details). In addition to the samples that span the duration of the mantle circulation models, we test the potential longevity of the Indian Ocean MORB signature by analysing three MORB samples from a Proto-Tethys ophiolite, dated at 550 Ma^32^ and two from a subsidiary basin of the early Pacific-Panthalassic basin – the Paleo-Asian Ocean ophiolite—dated at 600 Ma^33^.

## Results

The final configuration of all the new mantle circulation models were similar to each other, regardless of the plate motion history used, or duration. In each case, particles from *Tethyan-Indian* and *intra-Pacific* starting positions remained within the same hemisphere in which they started (Fig. 1). From each initial starting position, particles circulated to all depths of the mantle.

### Experiments with upper mantle starting positions

Those that started in the ‘upper mantle’ beneath *Tethys-Indian Ocean* rapidly (<20 Myrs) began to disperse towards the lowermost mantle (Fig. 1 and Supplementary Information Figs S4–S15). Particles after 119 Myrs in the **LBR** model were found predominantly at or near the core-mantle boundary (CMB) beneath Africa. In the **Seton** model, the particles after 200 Myrs were dispersed across the same geographic region and down to the CMB that they were in the **LBR** model, but they were somewhat more evenly distributed throughout the thickness of the mantle. Particles introduced beneath the *intra-Pacific* region in both the **LBR** and **Seton** models took longer to travel horizontally across the upper mantle before descending to mid- and lower mantle depths: this was a result of the larger size of the Pacific plate, and therefore farther distances to a subduction zone. In both the **LBR** and **Seton** models, particles introduced in the *intra-Pacific* region ended up at the CMB, but particles in the **Seton** model were more widely dispersed geographically around the CMB than those in the **LBR** model (*cf*. Supplementary Information Figs S6 and S7).

### Experiments with ‘mid-mantle’ starting positions

In experiments where the particles were introduced directly into the ‘mid-mantle’, particles spread both upwards and downwards: those introduced below the *Tethys-Indian Ocean* region predominantly ended up at the CMB beneath Africa (Supplementary Information Figs S8 and S9), whereas of those introduced beneath the *intra-Pacific* region a larger proportion were returned sooner to the upper mantle (Supplementary Information Figs S10 and S11) and then spent longer in the upper mantle before being recycled to greater depths. In this respect the **LBR** and **Seton** model results were similar, except that, of particles introduced below the Pacific in the **Seton** model, proportionally more ended up in the lower mantle, because of this model’s longer duration.

### Experiments with ‘lower mantle’ starting positions

In models where tracer particles were introduced directly into the ‘lower mantle’, we tried not to place particles directly in upwellings but, because of the smaller areas involved, could not completely avoid all upwelling and downwelling areas. ‘Lower mantle’ particles nevertheless failed to spread out ubiquitously around the CMB but instead returned to the upper mantle directly overhead, with lag-times ranging from >63 to ~20 Myrs. In the case of the **LBR** plate history, with particles introduced to the *Tethys-Indian Ocean* region, particles initially descended to cluster close to the CMB, before travelling upwards in thin sheets to the upper mantle, still beneath the *Tethys-Indian Ocean* (Supplementary Information Fig. S12). The thin sheets or ‘screens’ of rising tracers seemed to mark edges of the large convection cells. In the **Seton** model, a greater proportion of the particles introduced directly to lowermost mantle beneath the *Tethys-Indian Ocean*, moved up, away from the CMB in a shorter time-frame (Supplementary Information Fig. S13). With regards to the **LBR** *intra-Pacific* models, particles again pooled at the CMB before returning to the upper mantle beneath the Pacific. Interestingly, in the **Seton** models, with the longer duration than the LBR models, some particles appeared to complete the full cycle through the mantle, dispersing to the upper mantle within ~80 myrs before clustering close to the CMB by the end of the model run (Supplementary Information Figs S14 and S15).

### Geochemical validation

The mantle circulation models appear to show that the mantle convects from top to bottom as a whole, but laterally within two discrete domains. There appears to be minimal mixing or transfer of material laterally between the hemispherical divisions, and convection within the upper mantle is as much constrained by the division as the lower mantle. But is this really what we see from robust geochemical isotope data in the natural system? Here, we compare Hf-Nd isotopes for 32 carefully selected MORB samples against a discriminant for Indian versus Pacific isotope compositions (plotted on Fig. 2 as ΔεHf_i_, as defined within^30^; this notation is a deviation in Hf and Nd isotope space from a discrimination line between Indian- and Pacific MORB - Indian Ocean MORB has predominantly negative ΔεHf_i_ values, whereas Pacific MORB has positive values. See Fig. 2a for explanation). The discrimination line was determined from present-day compositions of Indian and Pacific MORB^30^.

### Tethyan-Indian MORB

Of the 19 MORB samples analysed from six Tethyan-Indian ophiolites, nearly all have negative values of ΔεHf_i_ (−4.98 to 2.06; Fig. 2), plotting very clearly on the Indian Ocean MORB side of the discrimination line (samples with positive ΔεHf_i_ are discussed below). The three 550 Myr Proto-Tethys MORB samples, all have high negative values of ΔεHf_i_ (−4.87 to −3.29) showing strong similarities with Tethyan-Indian Ocean MORB.

### Old Pacific MORB mantle

Of the 12 samples analysed from old Pacific MORB, almost all have positive ΔεHf_i_ values (−2.56 to 2.00; Fig. 2); just two have negative ΔεHf_i_ values (discussed below). Three samples analysed from the 600 Myr Paleo-Asian Ocean, have a wide range of ΔεHf_i_ values spanning from −1.56 to 8.58. The strongly positive values are the most positive values analysed in this study, but the heterogeneity of these Paleo-Asian Ocean basalts is in-keeping with Nd-Pb isotope data from 371 Myr old Paleo-Asian basalts from northern China^34^. The position of the Paleo-Asian Ocean is obviously poorly constrained at 600 Ma, but appears to have been some sort of junction between the Proto-Pacific and Proto-Tethys^35^.

### Old Atlantic MORB mantle

prior to the closure of the Tethys Ocean, but long before the full opening of the southern Atlantic, the central Atlantic Ocean was a small spreading basin. The only true MORB sample from the Central Atlantic at this time (~124 Ma) was analysed to determine whether it reflected a Tethyan-Indian-type signature, or a more Pacific-type influence. On εHf-εNd discrimination plots, *present-day* Atlantic MORB compositions straddle the Indian-Pacific boundary^14^, represent either a transitional mixture of the Indian – Pacific mantle compositions, or a unique reservoir with its own evolutionary history. The sample from the Central Atlantic gives a ΔεHf_i_ value of 0.06, consistent with present-day Atlantic MORB compositions. It is beyond the scope of this project to assess the origin of the Atlantic MORB composition.

### Exceptions within the Tethyan-Indian MORB samples – Ligurian MORB mantle

The only *Tethyan-Indian* MORB samples to not have negative ΔεHf_i_ values were amongst the samples from the Ligurian ophiolite in Italy; 5 of the 6 analysed samples from here have positive ΔεHf_i_ values up to 2.06. The Ligurian ophiolite is thought to represent crust that formed in a subsidiary basin at the western-most limit of Tethys, at a time when the Tethys Ocean to the east was closing. The straddling position of ΔεHf_i_ values of the Ligurian samples plot similar to values of the Central Atlantic sample and therefore could represent a mixture of Indian and Pacific MORB mantle *at the time* of Ligurian crust formation, or a longer-lived distinct ‘Atlantic-type’ mantle source that may or may not represent previous mixing of Indian and Pacific-type mantle. Discussion of this will not be within the remit of this paper.

### Exceptions within old Pacific MORB samples

Only two samples from old Pacific crust have negative ΔεHf_i_ values; one from 116 Ma NW Pacific with a value of −2.56 and one from 150 Ma SW Pacific with a value of −1.82. Rare, anomalous Indian-type compositions have been noted previously in the Pacific^36^ and similarly the other way around with anomalous occurrences of Pacific compositions within the Indian Ocean crust, e.g. Masirah^13^, and may represent minor heterogeneities within the upper mantle due to incomplete mixing. This will be discussed further below.

### Validation overview

The geochemical validation demonstrates that a clear distinction between Indian and Pacific upper mantle compositions extends all the way through the 200 Myrs as simulated in our mantle circulation models. As in the models, there has been an overwhelming dominance of two large (hemispheric) convection cells, and only small, localised domains (e.g. Liguria) where there has been some partial mixing or heterogeneity. We have, in addition for the first time used robust Hf-Nd isotope pairs, and this suggests that previous distinctions based on Pb isotopes^13^ are valid. Moreover, the new data extend the record of distinct Indian Ocean MORB compositions further back in time to 550 Ma (previously recognised at 371 Ma^34^), showing that differences between an Indian Ocean-type MORB and a Pacific-type MORB have existed throughout the Phanerozoic even though the global extents at that time remain to be mapped out. Determining the origin of the various MORB signatures is beyond the scope of the present study.

## Discussion

A stand-out result of the mantle circulation models is that, despite a level of variation between models, all the model runs showed that material in the upper mantle did not spread and mix ubiquitously, neither through descent to the lowermost depths of the mantle nor on its subsequent return flow to more shallow depths. Furthermore, this overall pattern was not diminished for the two alternative current plate motion history constructions, and their different duration times. More importantly, when descended lowermost mantle material returned to shallow levels, it returned to a similar *geographic* location from where it had been 100+ Myrs previously. This general conclusion wasn’t intuitively apparent prior to the study. Such a phenomena would enable significant geographic heterogeneities to persist for 100’s Myrs even in the upper mantle, as now validated by the Hf-Nd isotope data. The Hf isotope data, which is not affected by low-moderate temperature subduction-alteration, is entirely consistent with the mantle circulation models and demonstrates that the mantle below the present Indian Ocean could have maintained a distinct difference from that beneath the Pacific during plate re-organisations.

A limitation of all mantle circulation models is the lack of well-constrained plate reconstructions before 200 Ma, due to the loss of ocean crust from before that time. We designed the study to extend the significance of the results, by incorporating tracer particles initially positioned at mid- and lowermost mantle levels, as well as upper mantle (see Supplementary Information Part 1). The general results of our models show a dominantly up-down convective pattern with upper mantle ending up in the lower mantle – geographically below where it started – and lower mantle ending up in the upper mantle – geographically above where it started. Our models suggest transfer times through the mantle of <100 Myrs, and therefore speculate that particles started in the lowermost mantle could represent mantle that had convected from elsewhere, e.g. the upper mantle, <100 Myrs prior.

It seems that the overall pattern of convection adopted by these models is approximately degree-2, in response to the nature of the imposed plate motions. A degree-1 convective pattern, as suggested for some periods of Earth history, might have resulted during assembly of Pangaea but is considered to have not occurred younger than 250 Ma^37^. The **Seton** model, starting at 200 Ma includes the start of super-continent break-up. Prior to super-continent break-up subduction around the edge of Pangaea would have provided an effective curtain to full mantle mixing, only enhancing the potential for separation of two regions within the mantle. With evidence of the Indian Ocean MORB geochemical signature present in Paleo-Tethys^19^ back to 550 Ma (Fig. 2), we again speculate that a dominantly up-down convective pattern, with broad regions restricted by downwelling slabs, would provide a mechanism of physical separation throughout the Phanerozoic. Furthermore, if this process can be extrapolated to the beginning of the Phanerozoic, then given that mantle segregation had to have occurred long before 550 Ma, to allow for a distinct isotopic signature to have developed by this time, we see no reason why this process might not have been active since the start of plate tectonics. However, it is hoped that mantle circulation models, utilising longer plate motion histories being developed, will in the future be able to test this.

In all the *Tethys-Indian Ocean* models, regardless of the depth of introduction of particles or the plate motion history, particles spread towards the central or southern parts of the Atlantic (Fig. 1; Supplementary Information Figs S4, S5, S8, S9, S12, S13). This pattern of convection is consistent with geochemical evidence of Indian Ocean MORB-type chemistry and the DUPAL isotope anomaly in the present Southern Atlantic (e.g. refs 8, 11 and 12). Despite the modelled spread of tracer particles into the southern Atlantic, no particles travelled laterally to the Pacific region, nor, interestingly, into the northern Atlantic.

We noted that the particles initially placed in the *intra-Pacific* region generally did not leave the Pacific area (using either plate motion history reconstruction; Supplementary Information Figs S6, S7, S10, S11, S14, S15). However, on a more detailed scale the models reveal localised, complex interplay between circulating mantle and subducting slab. This was evident in the **Seton** models for the western margin of the Pacific (Supplementary Information Figs S7, S11 & S15). The effect is consistent with Indian Ocean MORB compositions that are recorded in parts of the present western Pacific basins^29^, which could result from alteration of slab vergence beneath the North Fiji and Lau basins.

At the scale of the models, it is difficult to assess whether a small number of particles take subsidiary, capricious excursions (see Supplementary Information Fig. S15 for example) departing from the dominant convection patterns. Such behaviour was noted, for example, in other 3D numerical models^22^. Such convective behaviour is supported by geochemical data, in the form of isolated occurrences of Indian Ocean-type MORB in areas away from the Indian Ocean, such as the Lomonosov ridge in the Arctic^36^ and in the Northwest Pacific^38^. It could apply also to a hitherto unexplained small occurrence of Pacific-MORB reported from Masirah in the northeast Indian Ocean^13^.

Overall, this study reveals that lateral, geographic heterogeneities are as important as radial (depth) layering in preserving and developing chemical heterogeneities within the mantle. The models and chemical evidence combined demonstrate that lateral segregations within the mantle, and a dominant large-scale convection pattern, can persist and match the long-term position of sinking plates; this is consistent with early speculations of an interplay between isotope anomalies and subduction patterns^7^. We propose that this process could have been a dominant feature of mantle convection ever since competent plates were able to descend deep into the mantle.

The conclusion that the mantle preserves heterogeneities through deep time and that it achieves this by quasi-steady planetary-scale convection, is robust in that it is consistent with evidence from elsewhere that use different assumptions, approaches, and geometric set-ups that differ from Earth. For example, models that do not incorporate geological plate-history reconstructions, or even present-day plate configurations, yielded results with some overall similar features, such as limits of convection cells defined by downwellings, as in some cuboid-shaped ‘box’ models^39, 40^, some 2-dimensional theoretical consideration^41^, and models that suggest a subduction control on the location of thermal and/or compositional anomalies, such as the regions interpreted as large low shear velocity provinces (LLSVP^42–44^). Long-term physical isolation of upper mantle regions by curtains of descending slab material provides, for the first time, a mechanism to explain large-scale chemical heterogeneities (e.g. refs 45 and 46), and how they may persist for 100’s millions of years.

### Summary

The results confirm a persistent global disparity in upper mantle composition that maps onto large hemispheric cells. The modelled pattern of convection provides a mechanism by which large regions of the mantle could evolve different isotope compositions, which would otherwise homogenise during convective mixing. Furthermore, for the first time, this work demonstrates a process that can account for the observed (e.g. ref. 9) differences in depleted end-member compositions and the long-recognised, but enigmatic, similarity between Indian and Tethyan MORBs^13, 20^. Regardless of the specific plate motion histories used in the modelling, our results suggest that a dominantly up-down motion of convection, restricted laterally by downwelling slabs, could have been a long-lived process resulting in large-scale lateral isolation of the mantle. This process is potentially as important as radial layering, within the mantle, at influencing long-term chemical evolution and could have led to different histories of isotope development, even in the upper mantle. Convection restricted by physical barriers of downwelling slabs could have existed throughout the Phanerozoic and potentially since the onset of modern plate tectonics.

## Methods

### 3D spherical numerical modelling

A prescribed initial temperature condition was used to calculate initial pressure and velocity fields. The boundary conditions for the model are: isothermal upper and lower boundary temperatures (300 K and 3000 K, respectively); and free-slip velocity at the core-mantle boundary. Radial variations in viscosity were prescribed from a reference value of 2 × 10^21^ Pa s (after^47^); where (1) the lithosphere = x10 higher than reference; (2) asthenosphere = reference value; and (3) lower mantle = x30 higher. Transition from upper to lower mantle was applied gradually between 500 and 1445 km depth, aiding computational stability (Supplementary Information Fig. S1). The upper-lower mantle viscosity ratio is broadly consistent with estimates from geophysical studies (e.g. refs 3 and 48).

Passive tracer particles were implemented using a Lagrangian particle tracking method where particles were advected using a second order Runge-Kutta method; this is the order warranted by the linear shape functions of TERRA^49, 50^.

### ‘Initial’ conditions

Model input parameters, which were not varied, are given in Supplementary Information Table S1. Traditionally, mantle models are permitted to run for sufficient time before analysis, to erase the influence of the initial conditions. In a model with constant heat inputs, this is beyond the point at which heat fluxes show no long-term trend. This situation is referred to as ‘statistically steady state’ (e.g. ref. 51). Due to the dynamic nature of models driven by plate motion history, the models in this study are not strictly ‘statistically steady state’; rather, we initially generate a ‘steady-state’ condition (here termed *pre-condition*) and then further condition the model with an initial plate stage (here termed *plate condition*) (details given in the Supplementary Information Part 1).

### Output, with passive particles

Using the mantle temperature condition from the *plate conditioning* stage, models were allowed to forward step through the 119 Myrs or 200 Myrs of plate motion history, running from the oldest stage to present day, with passive particles introduced into one of the six regions (summarised in Supplementary Information Table S2 and output displayed in detail in Supplementary Information Figs S4 to S15). We output the 3D temperature and particle distribution fields at the end of each plate stage. Particles were counted for each of the 128 radial model layers through the mantle profile and determined as percentage of volume (adjusted for decreasing cell volume with depth) (Supplementary Information Figs S4 to S15, parts D).

### Sample selection for geochemistry

MORB samples were selected from well-characterised greenschist facies ophiolites of Neo-Tethys and Paleo-Tethys. Newly collected hand specimens, along with some of the powders from published studies (Supplementary Information Table S3 & Supplementary Dataset), were analysed by inductively-coupled mass spectrometry (ICP-MS) for trace and rare earth element data to assess their MORB-like affinities. We used an Agilent 7500 s at the Open University, UK (following the standard procedure of Rogers *et al*.^52^) with a 2 SD external precision routinely better than 2.5% for elements heavier than Rb (Supplementary Dataset). Samples were selected from the least altered available material. Following strict criteria (Supplementary Information Part 2), some lava or dyke samples from each of the selected ophiolites were found to preserve MORB (*sensu stricto*) chemistry, and therefore reflects shallow asthenospheric mantle melting without the involvement of arc fluids, e.g. prior to arc/back-arc fluid enrichment.

### Isotope geochemistry

New ^176^Hf/^177^Hf and ^143^Nd/^144^Nd isotope analyses were undertaken on the rock specimens that proved to have MORB (*sensu stricto*) compositions following our assessment criteria (Supplementary Information Part 2).

Neodymium-Hf isotopes were prepared on powders that were first leached in 6 M HCl at 50–100 °C for between 30 mins and 1 hour. Samples were then spiked with mixed ^149^Sm-^150^Nd and ^176^Lu-^176^Hf spikes, and dissolved using a standard open-beaker HF-HNO_3_-HCl dissolution. Samples were carefully checked using a binocular microscope for any residual phases following dissolution, prior to elemental separation. Hafnium was separated using LN-SPEC resin, following the procedure of Münker *et al*.^53^. A REE concentrate was separated using Eichrom AG50X8 cation exchange resin, and Nd, Sm and Lu were then separated using LN-SPEC resin. Isotopic analyses were undertaken at the NERC Isotope Geosciences Laboratory (NIGL), UK. Hafnium isotopes were analysed on a Thermo Scientific Neptune Plus multi-collector (MC)-ICPMS. The data were corrected for mass fractionation during the run by normalisation to ^179^Hf/^177^Hf = 0.7325. Minimum uncertainties are derived from external precision of standard measurements for JMC475. Samples were run in several sessions over three years. Results for JMC475 run during each session are shown in Supplementary Information Table S4. Blanks were routine measured during these sessions and varied between 90 and 250 pg Hf. To allow for inter-laboratory bias, results are quoted relative to a preferred value for JMC475 of 0.282160^54^. Repeat analyses of BCR2 run with the samples gave a mean value of 0.282863 ± 0.000017 (1-sigma, n = 8).

Neodymium was run in multidynamic mode, as the metal species on double Re filaments using a Triton multi-collector mass spectrometer. The effects of fractionation during runs were eliminated by normalizing ^143^Nd/^144^Nd to a value of ^146^Nd/^144^Nd = 0.7219. Solution standards run with the samples over the three-year duration of the project gave results as follows: La Jolla, 0.511845 ± 0.000006 (1-sigma, n = 24); JNd-i, 0.512100 ± 0.000008 (1-sigma, n = 27). The offset from accepted values (La Jolla = 0.511860; JNd-i = 0.512115) is identical. Sample values for ^143^Nd/^144^Nd are reported relative to the accepted values of the standards run in each analytical session. Nine analyses of the BCR-2 rock standard analysed with the samples gave a mean ^143^Nd/^144^Nd ratio of 0.512637 ± 0.000009 (1-sigma). Blanks measured throughout this time varied between 30 and 200 pg Nd.

## Electronic supplementary material

Supplementary Information

Dataset 1
