# Supplementary material for: Whole-mantle convection with tectonic plates preserves long-term global patterns of upper mantle geochemistry
Source: Sci Rep. 2017 May 12;7:1870. doi: 10.1038/s41598-017-01816-y (PMC5431867; doi:10.1038/s41598-017-01816-y)
Supplement: Supplementary file 1 — Supplementary Information [file 41598_2017_1816_MOESM1_ESM.doc]

**Whole-mantle convection with tectonic plates preserves long-term global patterns of upper mantle geochemistry**

Barry, T.L.^1^, Davies, J.H.^2^, Wolstencroft, M.^3^, Millar, I.L.^4^, Zhao, Z.^5^, Jian, P.^6∞^, Safonova, I.^7,8^, Price, M.^2^

1. ^1^Department of Geology, University of Leicester, Leicester, LE1 7RH, UK.
2. ^2^School of Earth and Ocean Sciences, Cardiff University, Cardiff, CF10 3AT, Wales, UK.
3. ^3^JBA Risk Management, Broughton Hall, Skipton, North Yorkshire, BD23 3AE
4. ^4^NERC Isotope Geosciences Laboratory, British Geological Survey, Keyworth, Nottingham, NG12 5GG, UK

^5^School of Earth Science and Resources, China University of Geosciences, Beijing 100083, China

^6^Beijing SHRIMP Centre, Institute of Geology, Chinese Academy of Geological Sciences, Beijing 100037, China. ^∞^deceased

^7^Sobolev Institute of Geology and Mineralogy SB RAS, Novosibirsk 630090, Russia.

^8^Novosibirsk State University, Novosibirsk 630090, Russia

*Correspondence to tlb2@le.ac.uk

Supplementary Information

Part 1. Geodynamic modelling

Supplementary Information Fig. S1. The profile of the viscosity scaling factor versus depth (km); the reference value for viscosity was 2.0x10^21^ ^1^. The contrast in viscosity between the upper and lower mantle has been graded over a wide depth range, rather than an abrupt singular layer, which avoids computational instabilities.

Note: We avoided temperature dependent viscosity for computational reasons, given the resulting efficiency for solutions. However, from the work of Bello *et al.* ^2^ the details of large-scale flows on the timescale of a few hundred million years do not depend upon the degree of temperature dependent viscosity. Therefore, for the simulations presented here we have assumed that the canonical radial viscosity structure and plate history are the primary controls on large-scale geometry of flow, testing sensitivity by varying the mid-mantle viscosity jump and the plate history.  (NB: Temperature dependent viscosity would reinforce the dynamic blocking ability of colder, more viscous, subducted material. As such, the effects of temperature dependence would be expected to enhance, rather than reduce, the behaviour observed in this study).

Supplementary Information Table S1. Non-varying model input parameters

| Parameter | Value |
| --- | --- |
| Equation of state | Incompressible & Boussinesq |
| Reference density | 4500 kg m^-3^ |
| Reference viscosity  Gravitational acceleration | 2 x 10^21^ Pas  10 m s^-2^ |
| Vol. coefficient of thermal expansion | 2.5×10^-5^ K^-1^ |
| Thermal conductivity | 4 W m^-1^ K^-1^ |
| Specific heat (constant volume) | 1000 J K^-1^ kg^-1^ |
| Temperature at surface | 300 K |
| Temperature at CMB* | 3000 K |
| Radioactive heating | 4.000×10^-12^ W kg^-1^ |
| Inner radius of shell | 3.480×10^6^ m |
| Outer radius of shell | 6.370×10^6^ m |
| Rayleigh number | 1.976×10^8^ |


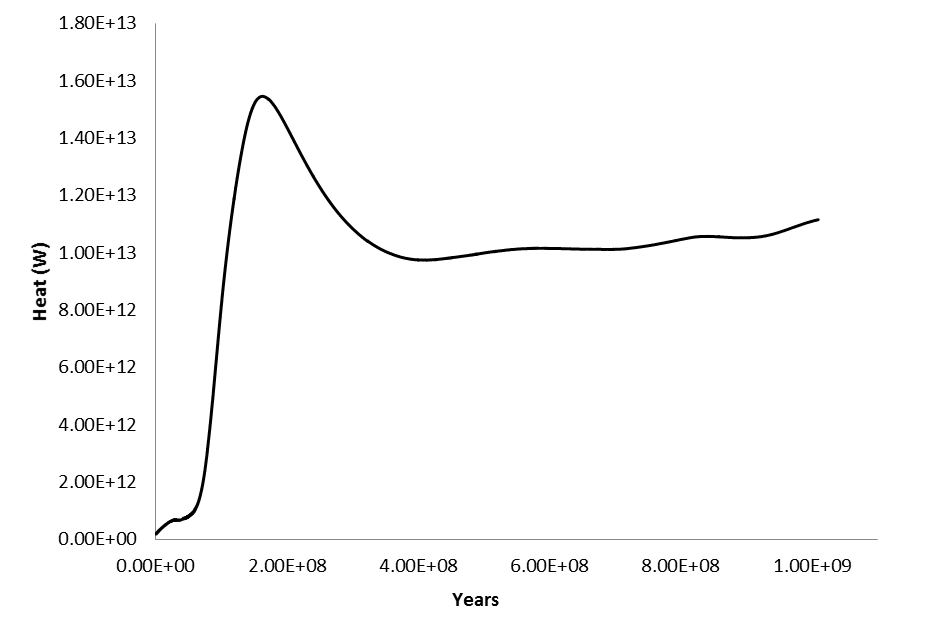


**A**


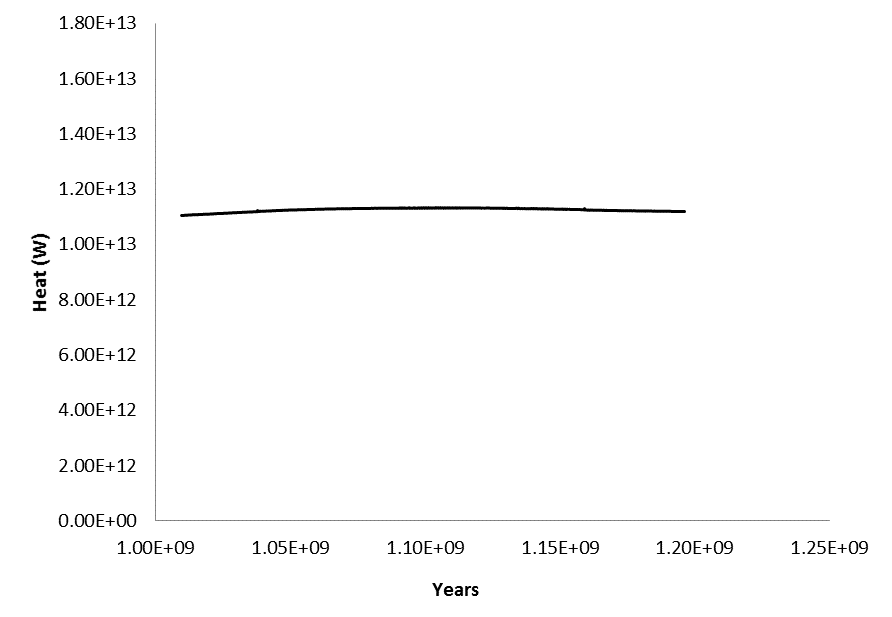


**B**

Supplementary Information Fig. S2. (a) Equivalent time elapsed (in years) versus surface heat flux (as a measure of input versus output) plotted to show how the model stabilised to a steady-state system in the pre-conditioning phase. (b) The same plot as above, but for the plate conditioning phase. During the plate conditioning, surface heat flux did not vary significantly.

*Pre-conditioning*

The condition of the mantle prior to the application of the plate history is particularly important as we aim to investigate the impact of time varying surface velocity inputs (the plate history) on the interior dynamics of the mantle. The true initial condition (at the very start of the model case) was a global temperature field with a conductive radial gradient and a free-slip upper surface. From this starting condition, the model was allowed to evolve to a state of stabilised heat output versus input. This process took 1.01x10^9^ iterations, equivalent to ~1.25 Gyrs time elapsed (Supplementary Information Fig. S2a).

*Plate conditioning*

Plate motion history imposes a specific geometry on the modelled mantle circulation different to the geometry that would be adopted in the absence of the plate velocities. Ideally we would have used a very long plate motion history (>500 Myr); however, these are not yet available in high temporal resolution (1-10Myr steps). Instead, we took the result at the end of *pre-conditioning*, and scaling the surface velocity to the average of the free-slip scenario, further evolved the model using the first (oldest) plate stage continuously for the equivalent of 150 Myrs. The duration of the plate conditioning phase was chosen to balance: (1) the temperature field not to resemble the starting condition i.e. that at the end of *pre-conditioning*, but (2) the structure of the temperature field not be dominated by the first plate stage. The *plate conditioning* stage produces the general geometry of convection in the presence of mobile surface plates, thus providing a ‘reasonable’ starting point for the plate motion histories. We derived the duration experimentally, by running the first plate stage continuously for the equivalent of 40, 80 and 150 Myr. The resulting heat input/output was found to not change from the end of pre-conditioning throughout the 150 Myrs equivalent time (Supplementary Information Fig. S2b) and temperature fields were plotted for comparison to one another and to the initial condition (Supplementary Information Fig. S3). We accept that the choice of plate conditioning time is likely to be somewhat subjective, as it is not possible to know the dynamic state of the mantle at either 119 Ma or 200 Ma at the start of the mantle circulation models.


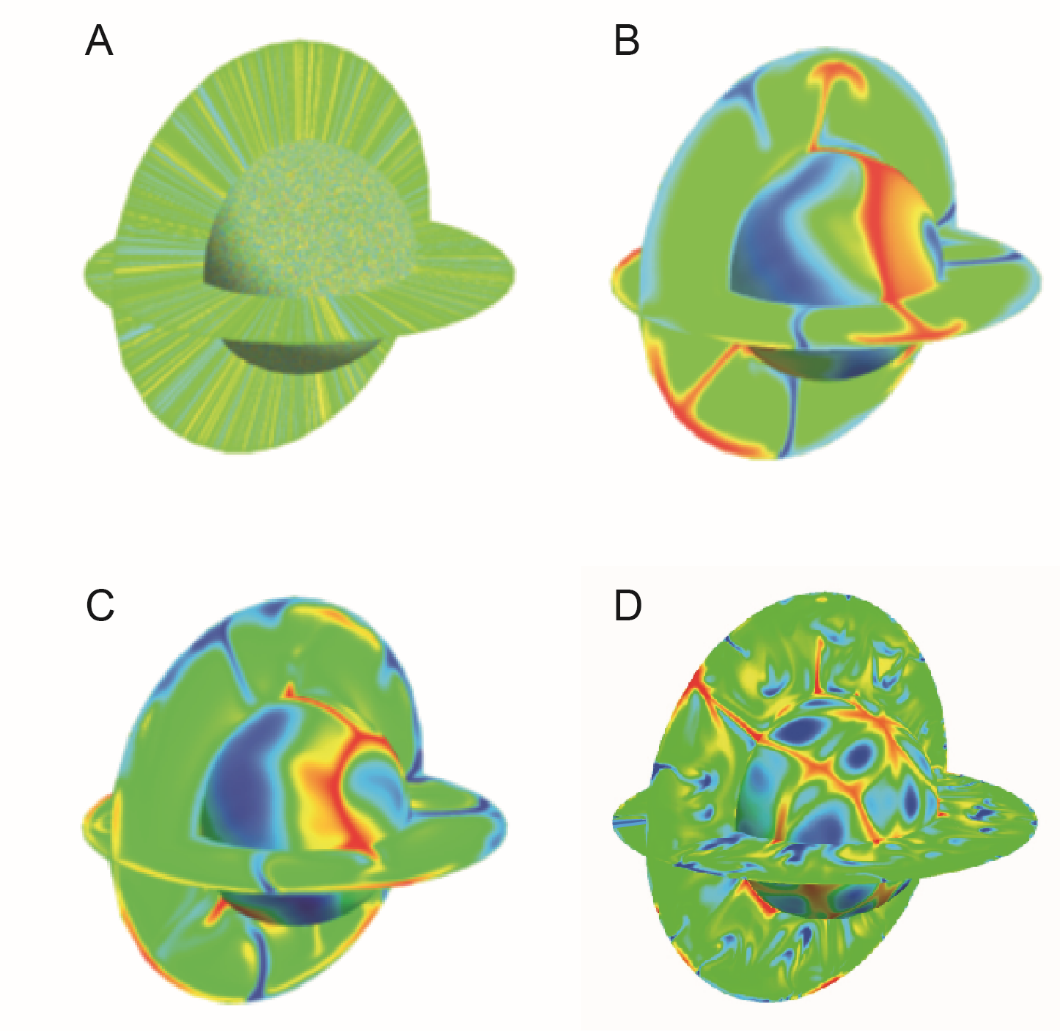


Supplementary Information Fig. S3. Plots of mantle temperature anomalies on the inner surface is just above the CMB. Temperature scale is arbitrary covering the full range of each case; blue represents colder and red hotter than average. (A) the initial condition used with all cases, (B) after 40 Myrs plate conditioning, (C) after 80 Myrs, and after (D) 150 Myrs.

Supplementary Information Table S2. Guide to Supplementary Information Figs S4 to S15. Note: continent outline is for present-day configuration and is only there as a guide for readers to locate relative positions through the different time and depth slices. The images in Figs S4 to S15 were all generated using visualisation software MantleVis; MantleVis is an in-house, unlicensed, distributed visualization tool written in C++ using the OpenGL API, and makes use of the OpenGL DisplayList tool (<http://pcwww.liv.ac.uk/~aeh/Software/MantleVis.htm> & <http://www.helix.cf.ac.uk/helix/?page_id=4>). Projections were selected to show all particles present on each layer.

|  |  |  |  |
| --- | --- | --- | --- |
|  |  |  |  |
|  |  | 119 Myrs history^3^ | 200 Myrs history^4^ |
|  |  |  |  |
| Upper mantle | Tethys-Indian Ocean | Supplementary Info Fig.S4 | Supplementary Info  Fig.S5 |
|  | Intra-Pacific | Supplementary Info Fig.S6 | Supplementary Info  Fig.S7 |
| Mid mantle | Tethys-Indian Ocean | Supplementary Info Fig.S8 | Supplementary Info  Fig.S9 |
|  | Intra-Pacific | Supplementary Info Fig.S10 | Supplementary Info Fig.S11 |
| Lower mantle | Tethys-Indian Ocean | Supplementary Info Fig.S12 | Supplementary Info Fig.S13 |
|  | Intra-Pacific | Supplementary Info Fig.S14 | Supplementary Info Fig.S15 |


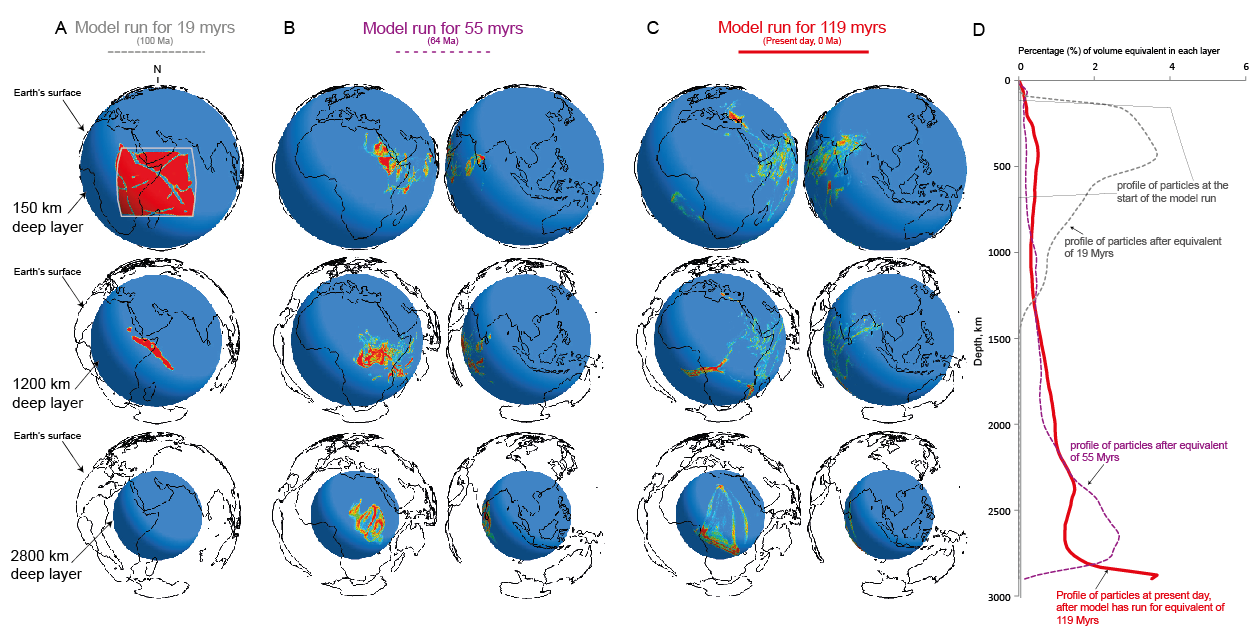


Supplementary Information Fig. S4. Initialisation of particles within the upper mantle (150-660 km) from the Tethys-Indian Ocean starting position, using the **LBR** model. Particle distributions are shown for 150 km, 1200 km and 2800 km depths for model at (A) 100 Ma, after 19 Myrs equivalent model circulation; (B) at 64 Ma, after 55 Myrs equivalent model circulation; and (C) at 0 Ma, when the model has run for the equivalent of 119 Myrs. Views in (A) are centred on the initial starting position of the particles (shown by grey box on 150 km panel) and do not exclude any particles outwith the view. Views in (B) have been selected to encompass the full spread of particles and similarly in (C). Note, none of the particles having started in the Tethys-Indian Ocean starting position spread to the Pacific or the Central Atlantic. Interestingly, the distribution of particles at 0 Ma in the lowermost mantle appears to be centred beneath Africa, as also observed in the 200 Myr mantle circulation models (Supplementary Information Fig. S5). (D) Percentage of particles in an equivalent volume in each layer plotted against depth (km). Due to the decreasing volume represented at individual layers in the spherical model, the population densities in the mapped views appear to lessen towards the lower mantle. Here, we plot the percentage of particles within individual layers equivalent to the volume of that layer (which also decreases with depth). Having started in the upper mantle layers, particles disperse towards deeper depths and after 55 Myrs (equivalent model time) particles have reached the core-mantle boundary (CMB). By the end of the model run the particles have dispersed to all depths but have a strong clustering in the lowermost mantle at the CMB. (Images generated using Mantlevis; see caption to Supplementary Information Table S2 for details).


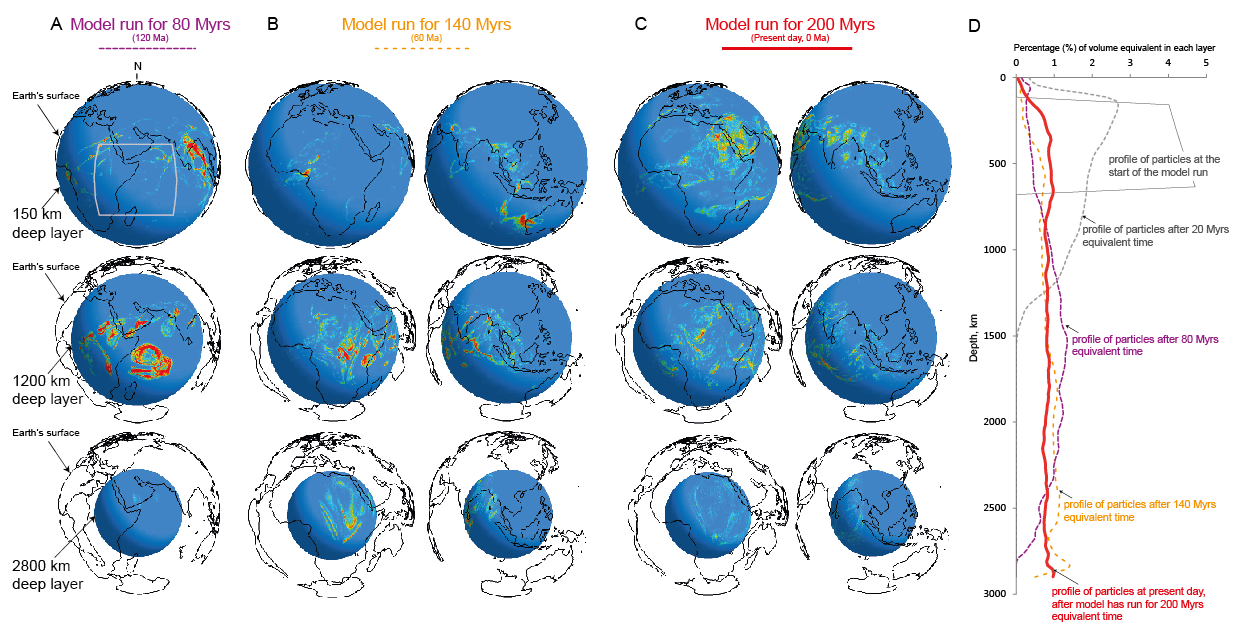


Supplementary Information Fig. S5. Initialisation of particles within the upper mantle (150-660 km), from the Tethys-Indian Ocean starting position, using the **Seton** model. Particle distributions are shown for 150 km, 1200 km and 2800 km depths for model at (A) 120 Ma, after 80 Myrs equivalent model circulation; (B) at 60 Ma, after 140 Myrs equivalent model circulation; and (C) at 0 Ma, when the model has run for the equivalent of 200 Myrs. Views in (A) are centred on the initial starting position of the particles (shown by grey box on 150 km panel) and do not exclude any particles outwith the view. Views in (B) have been given to encompass the full spread of particles and similarly in (C). Note, none of the particles having started in the Tethys-Indian Ocean starting position spread to the Pacific or the northern Atlantic. Interestingly, the distribution of particles in the lowermost mantle is centred beneath Africa, as seen in the models using **LBR** plate configuration (Supplementary Information Fig. S4). (D) Percentage of particles in an equivalent volume in each layer plotted against depth (km). Due to the decreasing volume represented at individual layers in the spherical model, the population densities in the mapped views appear to lessen towards the lower mantle. Here, we plot the percentage of particles within individual layers equivalent to the volume of that layer (which also decreases with depth). Having started in the upper mantle layers, particles rapidly disperse towards deeper depths (see profile at 20 Myrs), and after 80 Myrs (equivalent model time) particles have almost reached the core-mantle boundary. By the end of the model run the particles have dispersed to all depths quite evenly except in the uppermost (lithosphere) layers, but have not dispersed to any areas beyond the Mid-Atlantic Ridge, the Pacific Ocean or the far north or south. (Images generated using Mantlevis; see caption to Supplementary Information Table S2 for details).


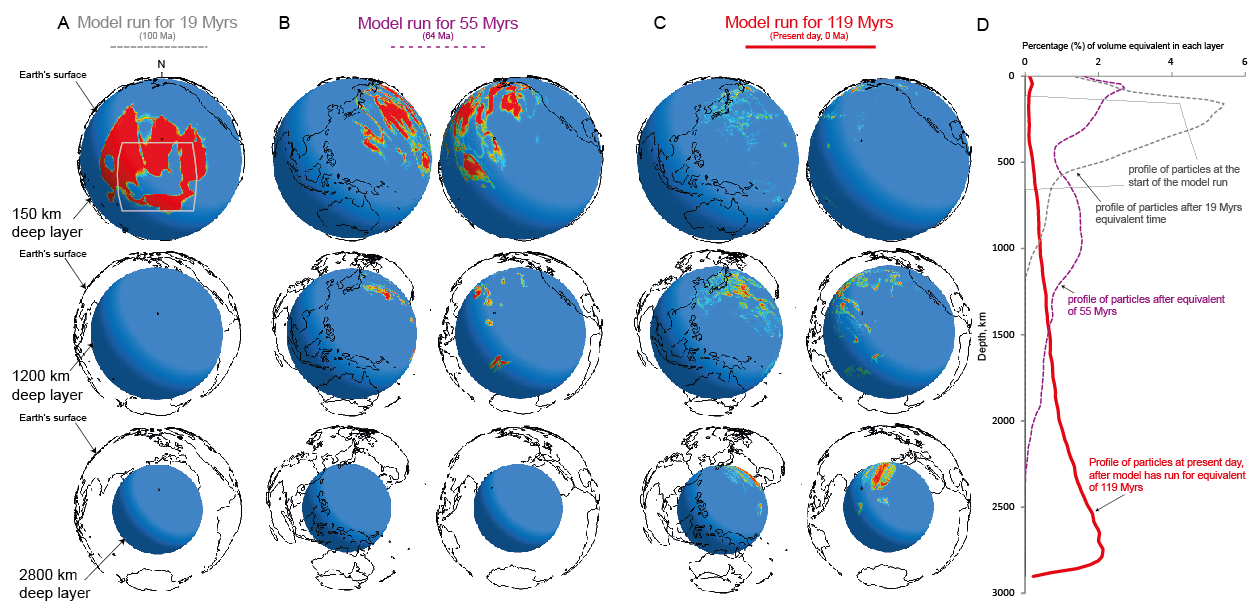


Supplementary Information Fig. S6. Initialisation of particles within the upper mantle (150-660 km), from the Pacific Ocean starting position, using the **LBR** model. Particle distributions are shown for 150 km, 1200 km and 2800 km depths for model at (A) 100 Ma, after 19 Myrs equivalent model circulation; (B) at 64 Ma, after 55 Myrs equivalent model circulation; and (C) at 0 Ma, when the model has run for the equivalent of 119 Myrs. Views in (A) are centred on the initial starting position of the particles (shown by grey box on 150 km panel) and do not exclude any particles outwith the view. Views in (B) have been selected to encompass the full spread of particles and similarly in (C). Note, none of the particles having started in the Pacific Ocean starting position spread to the Indian Ocean or beyond the eastern Pacific subduction zones. The distribution of particles in the lowermost mantle do not appear to mimic the broad swell of the LLSVP beneath the Pacific but are swept towards the north. (D) Percentage of particles in an equivalent volume in each layer plotted against depth (km). Due to the decreasing volume represented at individual layers in the spherical model, the population densities in the mapped views appear to lessen towards the lower mantle. Here, we plot the percentage of particles within individual layers equivalent to the volume of that layer (which also decreases with depth). Similar to Supplementary Information Fig. S4, the particles starting in the upper mantle disperse towards deeper depths but take slightly longer to reach the lowermost mantle than in the case of the Tethys-Indian Ocean. This is possibly due to the particles having to transit further, and therefore longer, in the upper mantle before reaching a subduction zone, to be returned to the deeper mantle. By the end of the model run, the particles dominantly distribute towards the lower mantle, but have not dispersed westwards beyond the western Pacific. (Images generated using Mantlevis; see caption to Supplementary Information Table S2 for details).


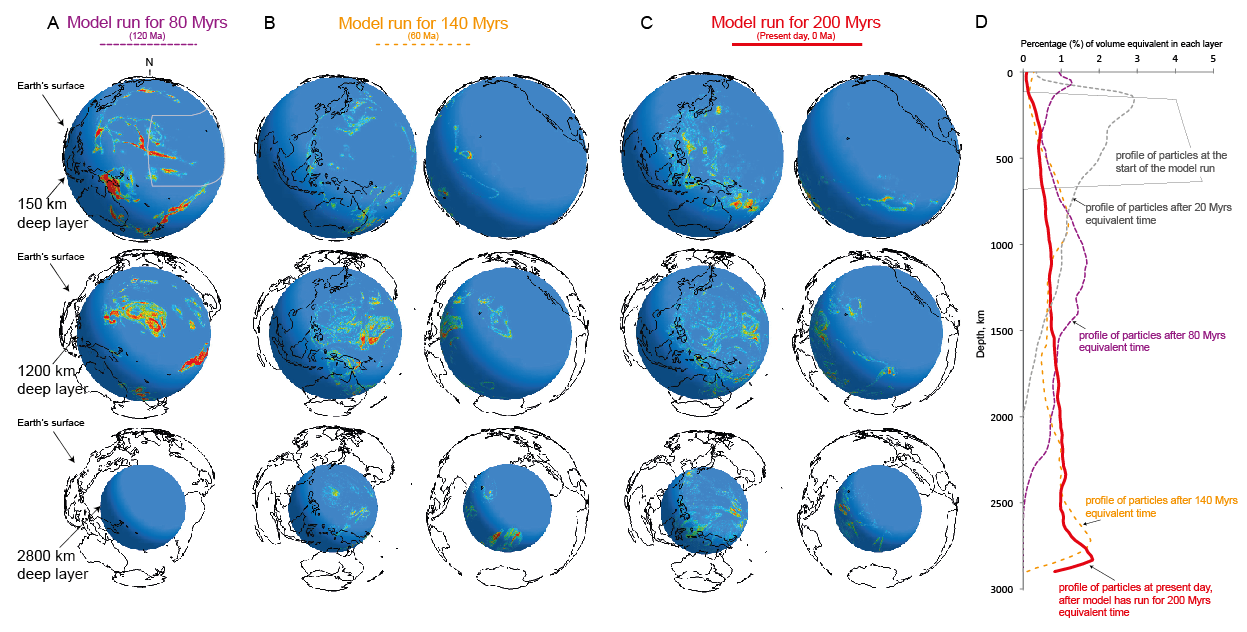


Supplementary Information Fig. S7. Initialisation of particles within the upper mantle (150-660 km), from the Pacific Ocean starting position, using the **Seton** model. Particle distributions are shown for 150 km, 1200 km and 2800 km depths for model at (A) 120 Ma, after 80 Myrs equivalent model circulation; (B) at 60 Ma, after 140 Myrs equivalent model circulation; and (C) at 0 Ma, when the model has run for the equivalent of 200 Myrs. Views in (A) are centred on the initial starting position of the particles (shown by grey box on 150 km panel) and do not exclude any particles outwith the view. Views in (B) have been given to encompass the full spread of particles and similarly in (C). Note, none of the particles having started in the Pacific Ocean starting area spread to the Indian Ocean or beyond the eastern Pacific subduction zones. The distribution of particles in the lowermost mantle does not appear to mimic the broad swell of the LLSVP beneath the Pacific but particles do appear to be swept towards the west and SW. (D) Percentage of particles in an equivalent volume in each layer plotted against depth (km). Due to the decreasing volume represented at individual layers in the spherical model, the population densities in the mapped views appear to lessen towards the lower mantle. Here, we plot the percentage of particles within individual layers equivalent to the volume of that layer (which also decreases with depth). Similar to Supplementary Information Fig. S5, the particles starting in the upper mantle rapidly disperse towards deeper depths (see profile at 20 Myrs – panel D). The particles take slightly longer to reach the lowermost mantle than in the case of the Tethys-Indian model (Supplementary Information Fig. S5), due to apparently spending a longer time in the upper parts of the lower mantle. By the end of the model run the particles dominantly distribute towards the deeper depths, but have not dispersed substantially westwards beyond the western Pacific margin. (Images generated using Mantlevis; see caption to Supplementary Information Table S2 for details).


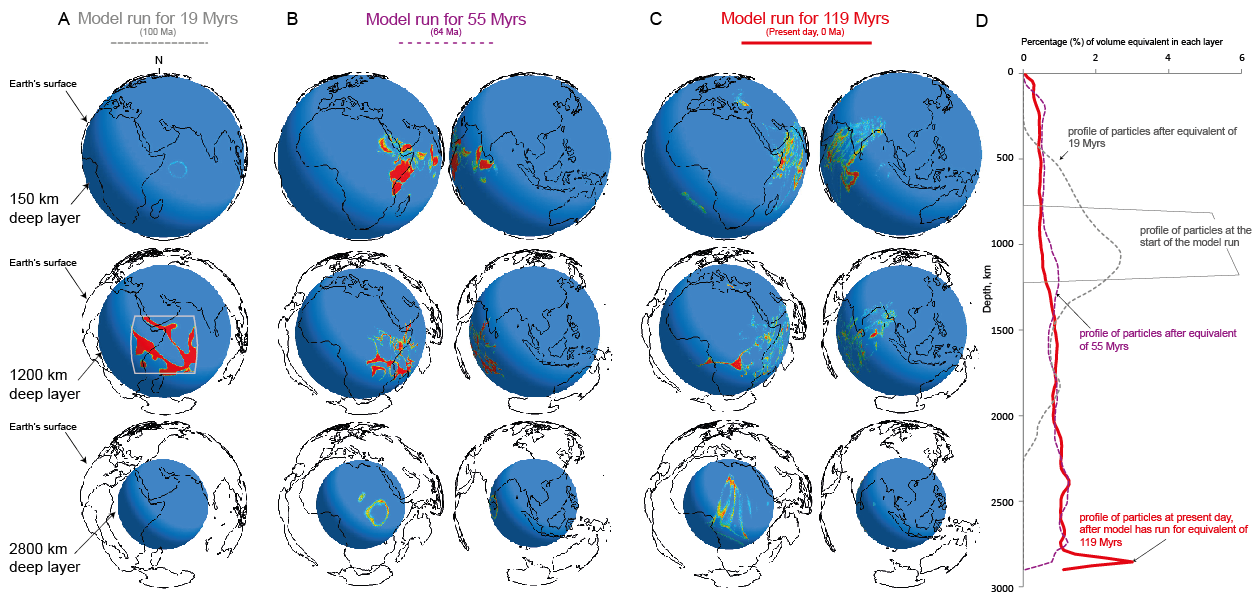


Supplementary Information Fig. S8. Initialisation of particles within the mid mantle (800-1200 km), from the Tethys-Indian Ocean starting position, using the **LBR** model. Particle distributions are shown for 150 km, 1200 km and 2800 km depths for model at (A) 100 Ma, after 19 Myrs equivalent model circulation; (B) at 64 Ma, after 55 Myrs equivalent model circulation; and (C) at 0 Ma, when the model has run for the equivalent of 119 Myrs. Views in (A) are centred on the initial starting position of the particles (shown by grey box on the 1200 km panel) and do not exclude any particles outwith the view. Views in (B) have been selected to encompass the full spread of particles and similarly in (C). Note, none of the particles having initialised in the Tethys-Indian Ocean starting position spread to the Pacific or the Central Atlantic. As with other models started in the Tethys-Indian Ocean position, the distribution of particles in the lowermost mantle is broadly similar to that seen in Supplementary Information Fig. S4. (D) Percentage of particles in an equivalent volume in each layer plotted against depth (km). Due to the decreasing volume represented at individual layers in the spherical model, the population densities in the mapped views appear to lessen towards the lower mantle. Here, we plot the percentage of particles within individual layers equivalent to the volume of that layer (which also decreases with depth). Having started in the mid mantle layers, particles spread towards the upper mantle and towards deeper depths and after the equivalent of 55 Myrs have reached all layers. By the end of the model run particles have dispersed to all depths but as with the upper mantle starting position (Supplementary Information Fig. S5) have a higher percentage at the base of the mantle. (Images generated using Mantlevis; see caption to Supplementary Information Table S2 for details).


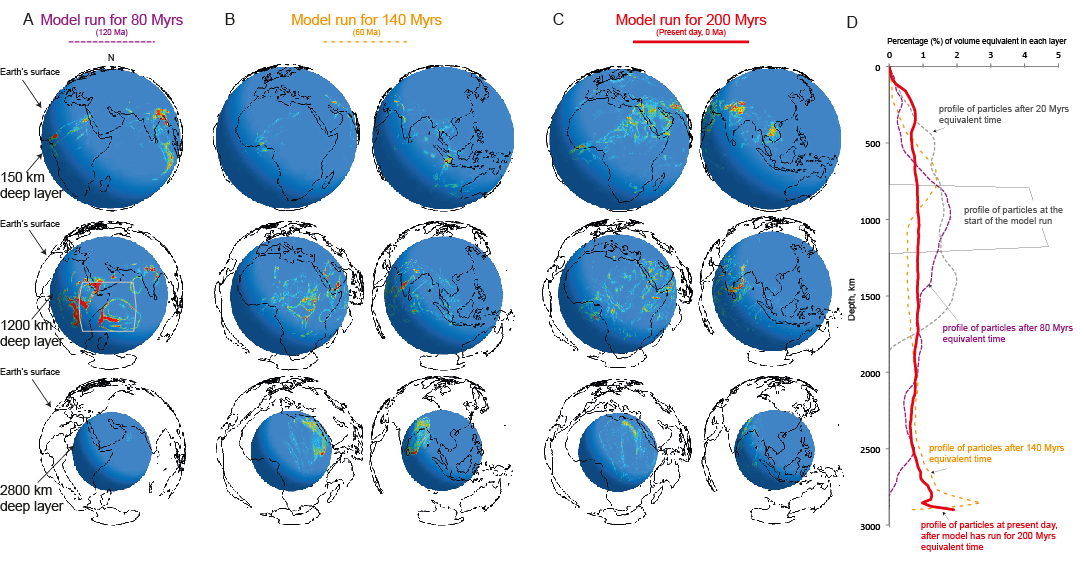


Supplementary Information Fig. S9. Initialisation of particles within the mid mantle (800-1200 km), from the Tethys-Indian Ocean starting position, using the **Seton** model. Particle distributions are shown for 150 km, 1200 km and 2800 km depths for model at (A) 120 Ma, after 80 Myrs equivalent model circulation; (B) at 60 Ma, after 140 Myrs equivalent model circulation; and (C) at 0 Ma, when the model has run for the equivalent of 200 Myrs. Views in (A) are centred on the initial starting position of the particles (shown by grey box on the 1200 km panel) and do not exclude any particles outwith the view. Views in (B) have been given to encompass the full spread of particles and similarly in (C). Note, none of the particles having started in the Tethys-Indian Ocean starting area spread to the Pacific or the Central Atlantic. And, as with the results in Supplementary Information Fig. S5, S6 and S8, the end distribution of particles in the lowermost mantle is broadly centred beneath Africa. (D) Percentage of particles in an equivalent volume in each layer plotted against depth (km). Due to the decreasing volume represented at individual layers in the spherical model, the population densities in the mapped views appear to lessen towards the lower mantle. Here, we plot the percentage of particles within individual layers equivalent to the volume of that layer (which also decreases with depth). Having started in the mid mantle layers, particles spread towards the upper mantle and towards deeper depths and after 80 Myrs (equivalent model time) have almost reached all layers. By the end of the model run particles have dispersed to all depths but have a higher percentage at the base of the mantle. (Images generated using Mantlevis; see caption to Supplementary Information Table S2 for details).


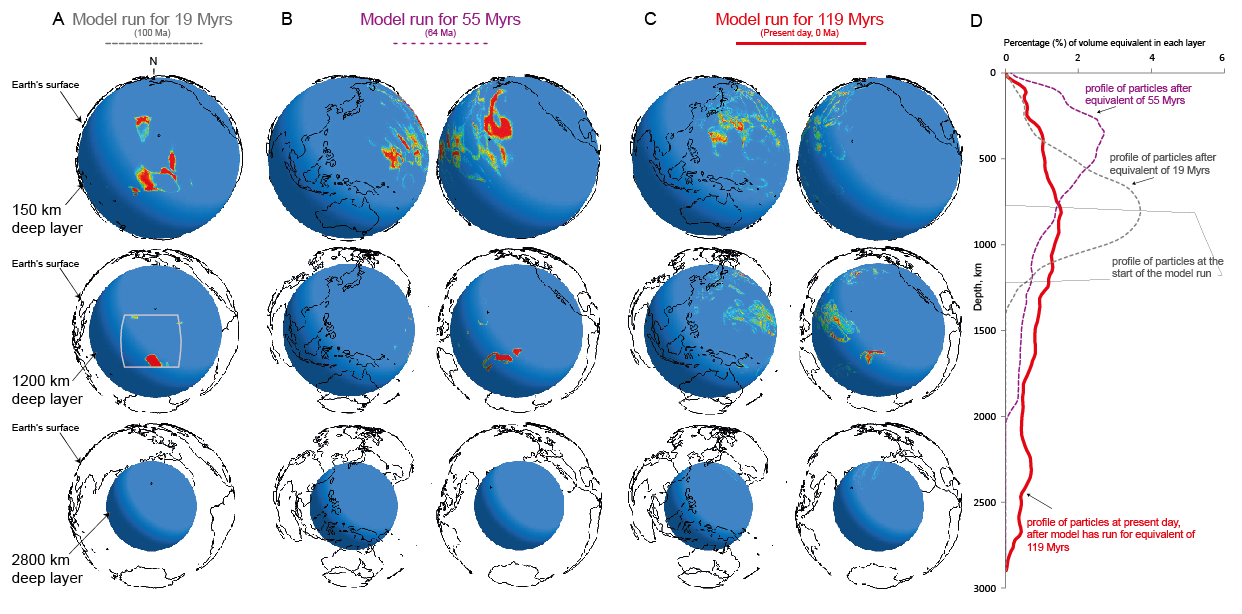


Supplementary Information Fig. S10. Initialisation of particles within the mid mantle (800-1200 km), from the Pacific Ocean starting position, using the **LBR** model. Particle distributions are shown for 150 km, 1200 km and 2800 km depths for model at (A) 100 Ma, after 19 Myrs equivalent model circulation; (B) at 64 Ma, after 55 Myrs equivalent model circulation; and (C) at 0 Ma, when the model has run for the equivalent of 119 Myrs. Views in (A) are centred on the initial starting position of the particles (shown by grey box on the 1200 km panel) and do not exclude any particles outwith the view. Views in (B) have been given to encompass the full spread of particles and similarly in (C). Note, none of the particles having started in the Pacific Ocean starting area have dispersed westwards towards the Indian Ocean or beyond the eastern Pacific subduction zones. (D) Percentage of particles in an equivalent volume in each layer plotted against depth (km). Due to the decreasing volume represented at individual layers in the spherical model, the population densities in the mapped views appear to lessen towards the lower mantle. Here, we plot the percentage of particles within individual layers equivalent to the volume of that layer (which also decreases with depth). The particles starting in the mid mantle disperse most dominantly towards the upper mantle but after the equivalent of 119 Myrs mantle circulation particles have distributed to all depths, with a slight higher percentage population in the mid mantle. (Images generated using Mantlevis; see caption to Supplementary Information Table S2 for details).


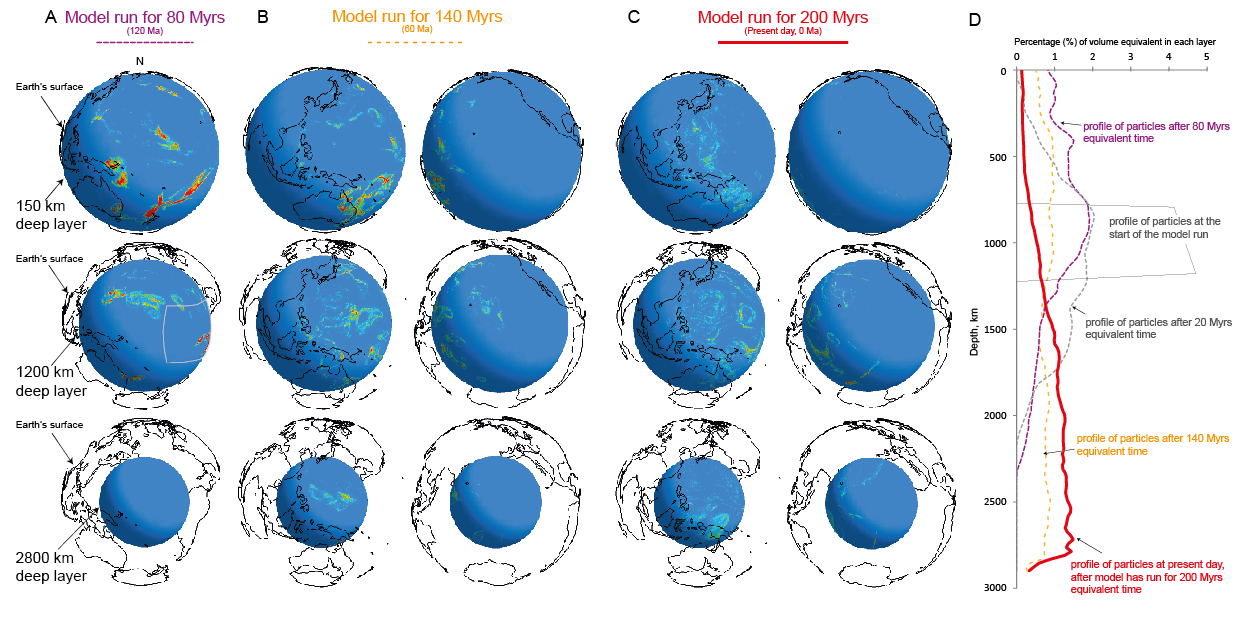


Supplementary Information Fig. S11. Initialisation of particles within the mid mantle (800-1200 km), from the Pacific Ocean starting position, using the **Seton** model. Particle distributions are shown for 150 km, 1200 km and 2800 km depths for model at (A) 120 Ma, after 80 Myrs equivalent model circulation; (B) at 60 M,a after 140 Myrs equivalent model circulation; and (C) at 0 Ma, when the model has run for the equivalent of 200 Myrs. Views in (A) are centred on the initial starting position of the particles (shown by grey box on the 1200 km panel) and do not exclude any particles outwith the view. Views in (B) have been given to encompass the full spread of particles and similarly in (C). Note, none of the particles having started in the Pacific Ocean starting area have dispersed westwards beyond the Izu-Bonin-Mariana arcs into the Indian Ocean or beyond the eastern Pacific subduction zones. The distribution of particles in the lowermost mantle does not appear to mimic the broad swell of the LLSVP beneath the Pacific though there is a cluster of particles towards the SW. (D) Percentage of particles in an equivalent volume in each layer plotted against depth (km). Due to the decreasing volume represented at individual layers in the spherical model, the population densities in the mapped views appear to lessen towards the lower mantle. Here, we plot the percentage of particles within individual layers equivalent to the volume of that layer (which also decreases with depth). Similar to Supplementary Information Fig. S10, the particles starting in the mid mantle disperse towards the upper mantle and to deeper depths though as in Supplementary Information Fig. S10 this does not appear to occur as rapidly as it does in the Tethys-Indian Ocean cases (Supplementary Information Fig. S8 and S9). By the end of the model run the particles dominantly distribute towards the deeper depths. (Images generated using Mantlevis; see caption to Supplementary Information Table S2 for details).


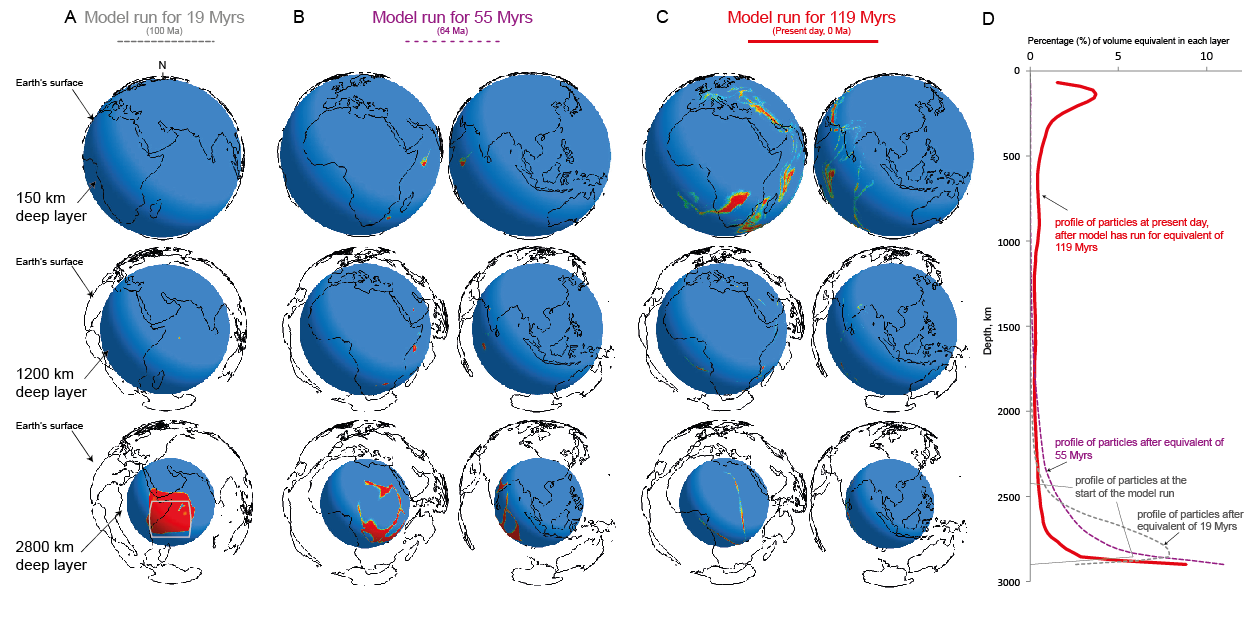


Supplementary Information Fig. S12. Initialisation of particles within the lowermost mantle (2450-2890 km), from the Tethys-Indian Ocean starting position, using the **LBR** model. Particle distributions are shown for 150 km, 1200 km and 2800 km depths for model at (A) 100 Ma, after 19 Myrs equivalent model circulation; (B) at 64 Ma, after 55 Myrs equivalent model circulation; and (C) at 0 Ma, when the model has run for the equivalent of 119 Myrs. Views in (A) are centred on the initial starting position of the particles (shown by grey box on the 2800 km panel) and do not exclude any particles outwith the view. Views in (B) have been given to encompass the full spread of particles and similarly in (C). Note, none of the particles having started in the Tethys-Indian Ocean starting area spread to the Pacific or the Central Atlantic. Interestingly, in all the Tethys-Indian Ocean models using the LBR plate configurations, particles appear to spread towards the southern Atlantic. (D) Percentage of particles in an equivalent volume in each layer plotted against depth (km). Due to the decreasing volume represented at individual layers in the spherical model, the population densities in the mapped views appear to lessen towards the lower mantle. Here, we plot the percentage of particles within individual layers equivalent to the volume of that layer (which also decreases with depth). Having started in the lowermost mantle layers, the particles do not spread out laterally around the CMB even though at first the particles do appear to populate the lowermost layers (see profile at 55 Myrs). Between 64 Ma and present day, particles are then returned to the upper mantle, focussed around the Tethys-Indian Ocean areas and the southern Atlantic. (Images generated using Mantlevis; see caption to Supplementary Information Table S2 for details).


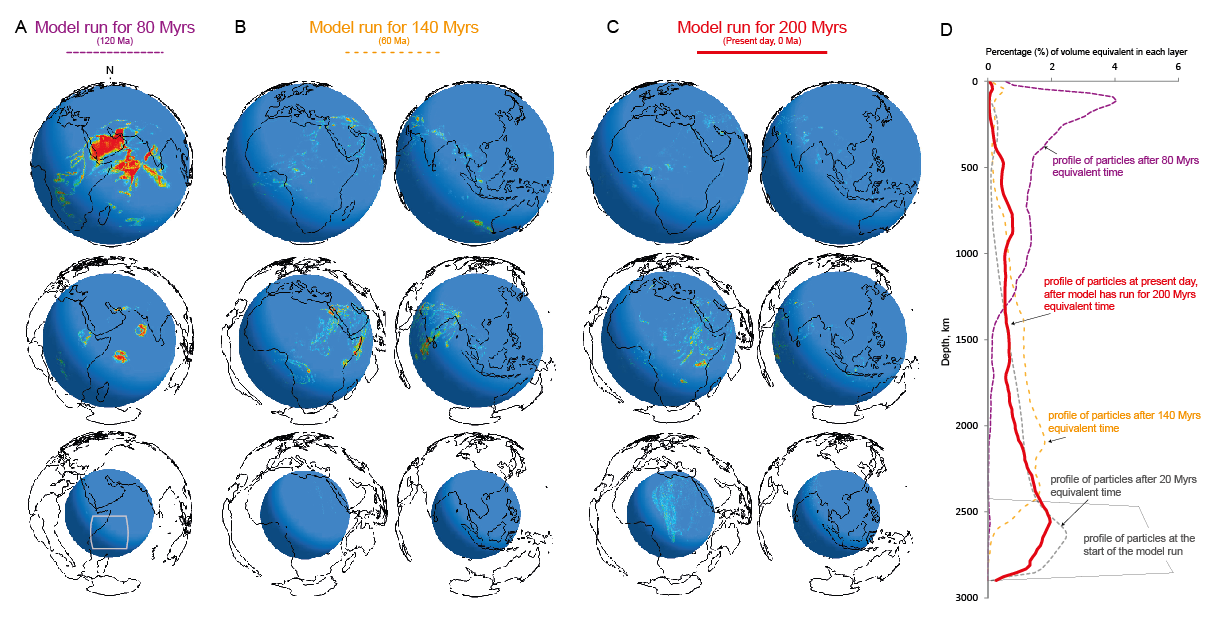


Supplementary Information Fig. S13. Initialisation of particles within the lowermost mantle (2450-2890 km), from the Tethys-Indian Ocean starting position, using the **Seton** model. Particle distributions are shown for 150 km, 1200 km and 2800 km depths for model at (A) 120 Ma, after 80 Myrs equivalent model circulation; (B) at 60 Ma, after 140 Myrs equivalent model circulation; and (C) at 0 Ma, when the model has run for the equivalent of 200 Myrs. Views in (A) are centred on the initial starting position of the particles (shown by grey box on the 2800 km panel) and do not exclude any particles outwith the view. Views in (B) have been given to encompass the full spread of particles and similarly in (C). Note, none of the particles having started in the Tethys-Indian Ocean starting area spread to the Pacific or the northern Atlantic. As with results from the other Tethys-Indian Ocean starting location models (Supplementary Information Fig. S5 & S9), the distribution of particles at 0 Ma in the lowermost mantle is broadly similar to the African LLSVP. (D) Percentage of particles in an equivalent volume in each layer plotted against depth (km). Due to the decreasing volume represented at individual layers in the spherical model, the population densities in the mapped views appear to lessen towards the lower mantle. Here, we plot the percentage of particles within individual layers equivalent to the volume of that layer (which also decreases with depth). Having started in the lowermost mantle layers, the particles do not spread out laterally around the CMB but instead disperse first to the upper mantle (see 80 Myrs distribution) and then more generally throughout all the depths. Within 140 Myrs particles have returned to the lower mantle again. (Images generated using Mantlevis; see caption to Supplementary Information Table S2 for details).


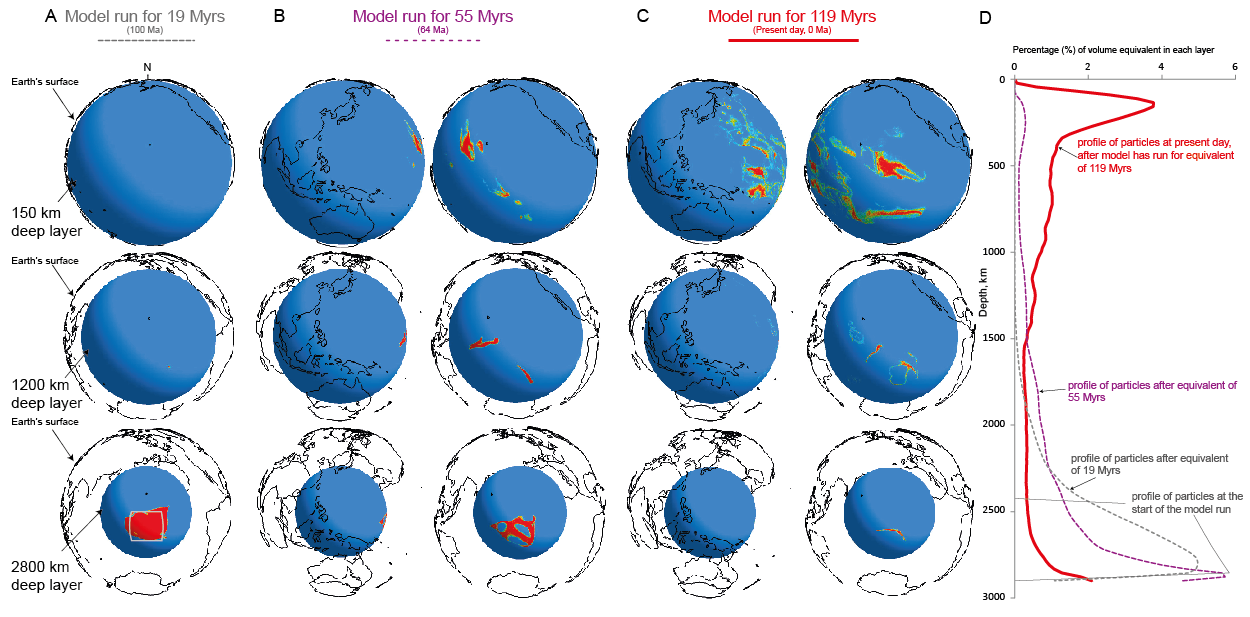


Supplementary Information Fig. S14. Initialisation of particles within the lowermost mantle (2450-2890 km), from the Pacific Ocean starting position, using the **LBR** model. Particle distributions are shown for 150 km, 1200 km and 2800 km depths for model at (A) 100 Ma, after 19 Myrs equivalent model circulation; (B) at 64 Ma, after 55 Myrs equivalent model circulation; and (C) at 0 Ma, when the model has run for the equivalent of 119 Myrs. Views in (A) are centred on the initial starting position of the particles (shown by grey box on the 2800 km panel) and do not exclude any particles outwith the view. Views in (B) have been given to encompass the full spread of particles and similarly in (C). Note, none of the particles having started in the Pacific Ocean starting area have dispersed westwards towards the Indian Ocean or beyond the eastern Pacific subduction zones. The distribution of particles in the lowermost mantle does not appear to mimic the broad swell of the LLSVP beneath the Pacific. (D) Percentage of particles in an equivalent volume in each layer plotted against depth (km). Due to the decreasing volume represented at individual layers in the spherical model, the population densities in the mapped views appear to lessen towards the lower mantle. Here, we plot the percentage of particles within individual layers equivalent to the volume of that layer (which also decreases with depth). Similar to Supplementary Information Fig. S13, the particles starting in the lowermost mantle collect near the base of the mantle, but instead of spreading out laterally around the CMB within the 119 Myrs of mantle circulation modelling, they return to the upper mantle, but only within the Pacific region. (Images generated using Mantlevis; see caption to Supplementary Information Table S2 for details).


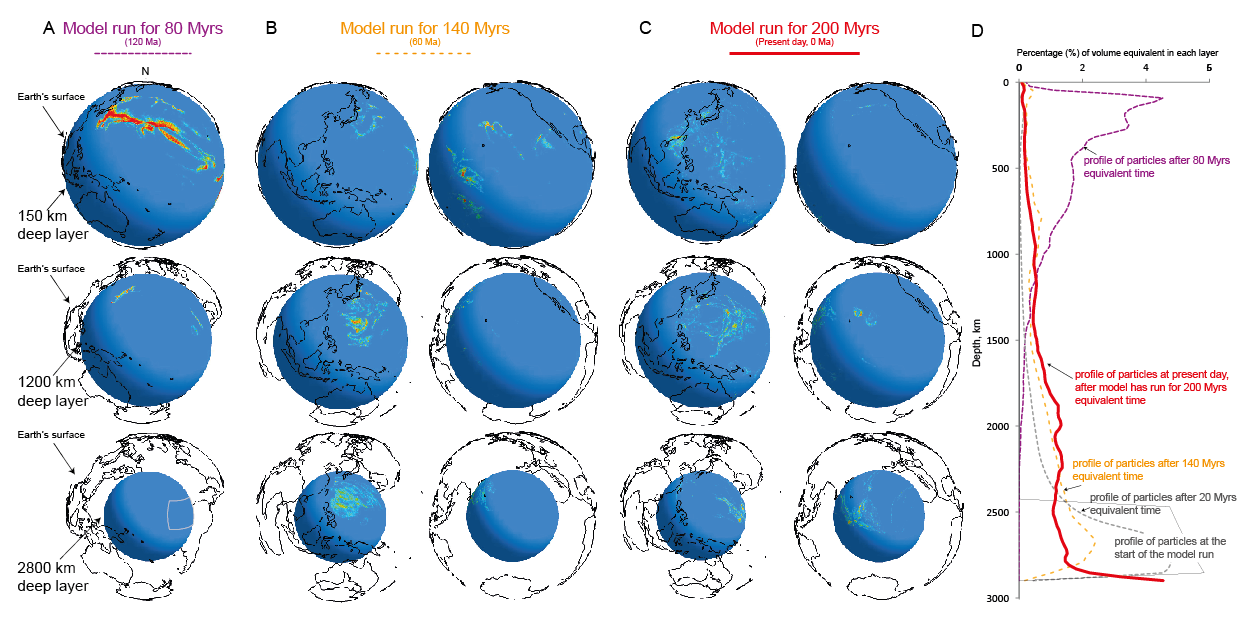


Supplementary Information Fig. S15. Initialisation of particles within the lowermost mantle (2450-2890 km), from the Pacific Ocean starting position, using the **Seton** model. Particle distributions are shown for 150 km, 1200 km and 2800 km depths for model at (A) 120 Ma, after 80 Myrs equivalent model circulation; (B) at 60 Ma, after 140 Myrs equivalent model circulation; and (C) at 0 Ma, when the model has run for the equivalent of 200 Myrs. Views in (A) are centred on the initial starting position of the particles (shown by grey box on the 2800 km panel) and do not exclude any particles outwith the view. Views in (B) have been given to encompass the full spread of particles and similarly in (C). Note, none of the particles having started in the Pacific Ocean starting area have dispersed westwards beyond the Izu-Bonin-Mariana arcs into the Indian Ocean or beyond the eastern Pacific subduction zones. The distribution of particles in the lowermost mantle does not appear to mimic the broad swell of the LLSVP beneath the Pacific. (D) Percentage of particles in an equivalent volume in each layer plotted against depth (km). Due to the decreasing volume represented at individual layers in the spherical model, the population densities in the mapped views appear to lessen towards the lower mantle. Here, we plot the percentage of particles within individual layers equivalent to the volume of that layer (which also decreases with depth). Similar to Supplementary Information Fig. S14, the particles starting in the lowermost mantle disperse towards the upper mantle within 80 myrs and then return to the lower mantle within 140 myrs. Unlike the Tethys-Indian Ocean case, particles appear to cluster very close to the CMB (below the depth shown in panel C) by the end of the model run. **Black boxes highlight possible particle excursions (see main text).** (Images generated using Mantlevis; see caption to Supplementary Information Table S2 for details).

Part 2. Geochemistry

Supplementary Information Table S3. Ophiolites used in this study and key references for sample material.

| Ophiolite locality | Main publications describing samples/ophiolite | Samples received from |
| --- | --- | --- |
| Alexander Island, Antarctica | Thomson & Tranter, 1986^5^ | I.L. Millar (NIGL, UK) |
| Mirdita, Albania | Dilek et al., 2008^6^ | M. Maffione (Utrecht, Netherlands). |
| Liguria, Italy | Montanini et al., 2008^7^; Rampone et al., 1998^8^; Sanfilippo & Tribuzio, 2011^9^ | R. Tribuzio (Padua, Italy). |
| SW Pacific crust, Site 91 | Saunders, 1987^10^ | A. Saunders (Leicester, UK) |
| Muslim Bagh, Pakistan | Khan et al., 2007^11^ | A. Kerr (Cardiff, UK) |
| Nidar, India | Ahmad et al., 2008^12^ | N. Harris (OU, UK) |
| Xigaze, Tibet | Niu et al., 2006^13^ | Z. Zhao (CUGB, China) |
| Central Atlantic | Burky, 1978^14^ | IODP request 21920A |
| NW Pacific, Site 86&88 |  | A. Saunders (Leicester, UK) |
| Venezuela | Beccaluva et al., 1996^15^; Giunta et al., 2002^16^ | A. Kerr (Cardiff, UK) |
| Semail, Oman | Godard et al., 2006^17^ | M. Godard (Montpellier, France) |
| Kurai, Russia | Safonova et al., 2008^18^ | I. Safonova (Novosibirsk, Russia) |
| Yushigou, China | Shi et al., 2004^19^; Hou et al., 2006^20^ | Z. Zhao (CUGB, China) |

Geochemical assessment criteria

Using geochemical data presented in Supplementary Dataset, sample compositions were assessed for their likeness to MORB composition (*sensu stricto*). We used the following criteria:

(1) Ti- Zr-Y discrimination diagram (after ^21^; Supplementary Information Fig. S16a);

(2) Th/Yb vs Nb/Yb and TiO_2_/Yb vs Nb/Yb (after ^22^; Supplementary Information Fig. S16b&c);

(3) Zr/Y versus Nb/Y (after ^23^; Supplementary Information Fig. S16d) and

(4) MORB-like multi-element distribution plots (Supplementary Information Fig. S16e).

Only samples that achieved all, or at least three of these criteria were included in the isotope study (see below).


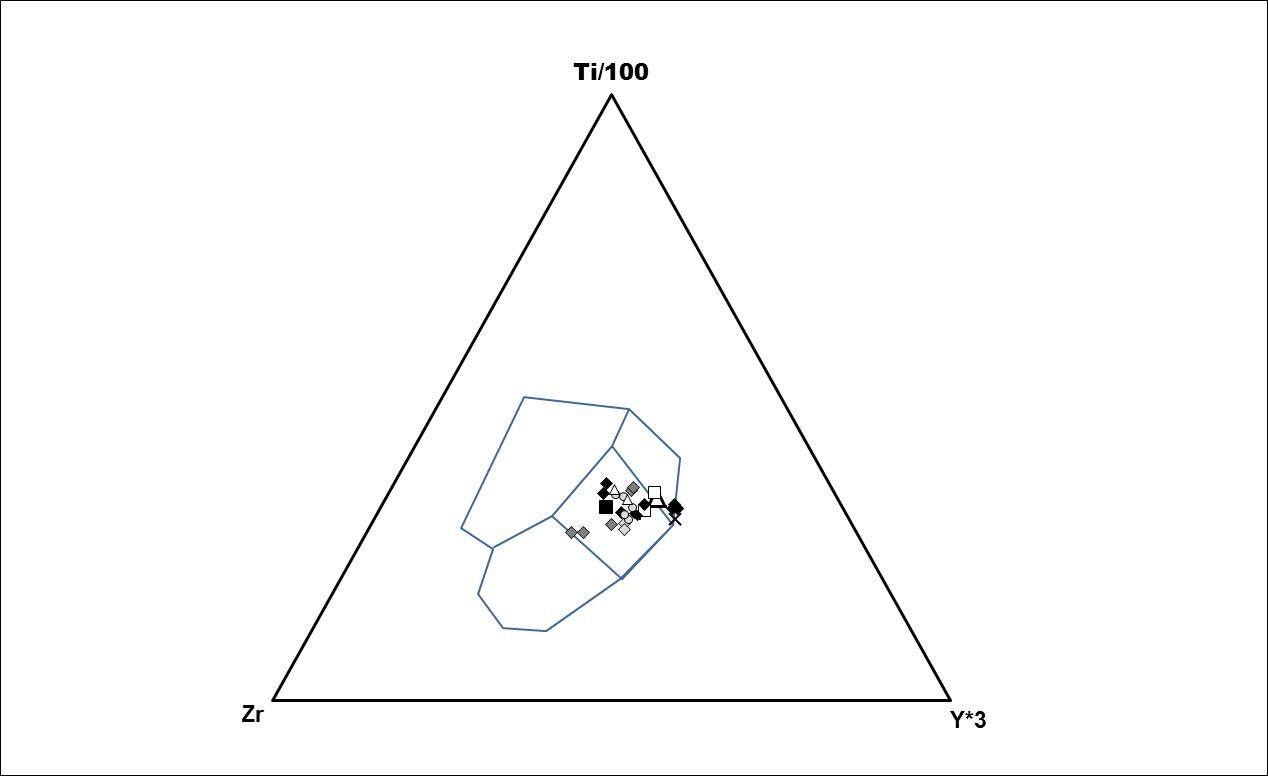


within-plate basalts

low-K arc tholeiites

ocean-floor basalts

calc-alkaline basalts

Supplementary Information Fig. S16(a). Zr-Ti-Y triplot (after ^21^) showing ophiolite samples plotting within the MORB field (symbols the same as for Figure 1, and below).


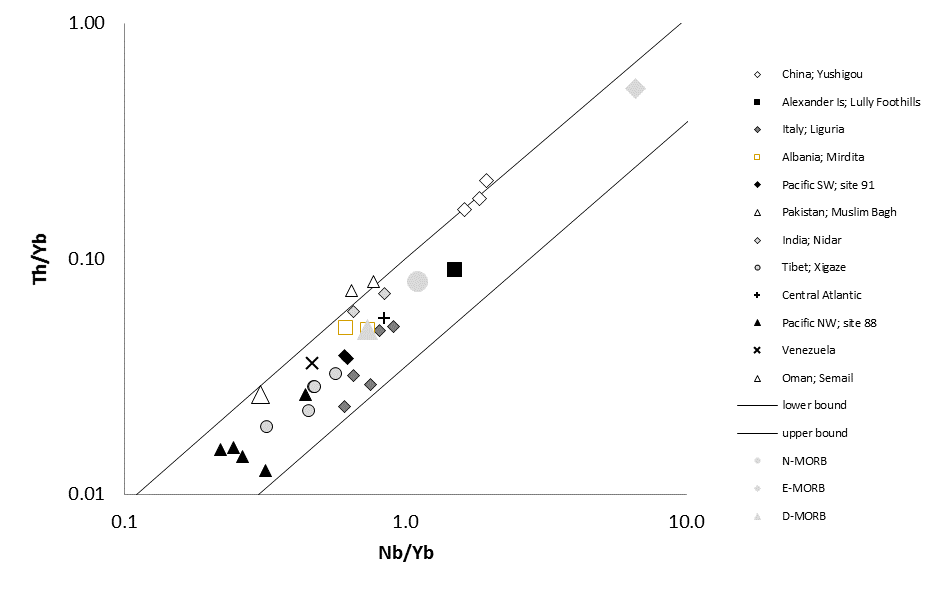


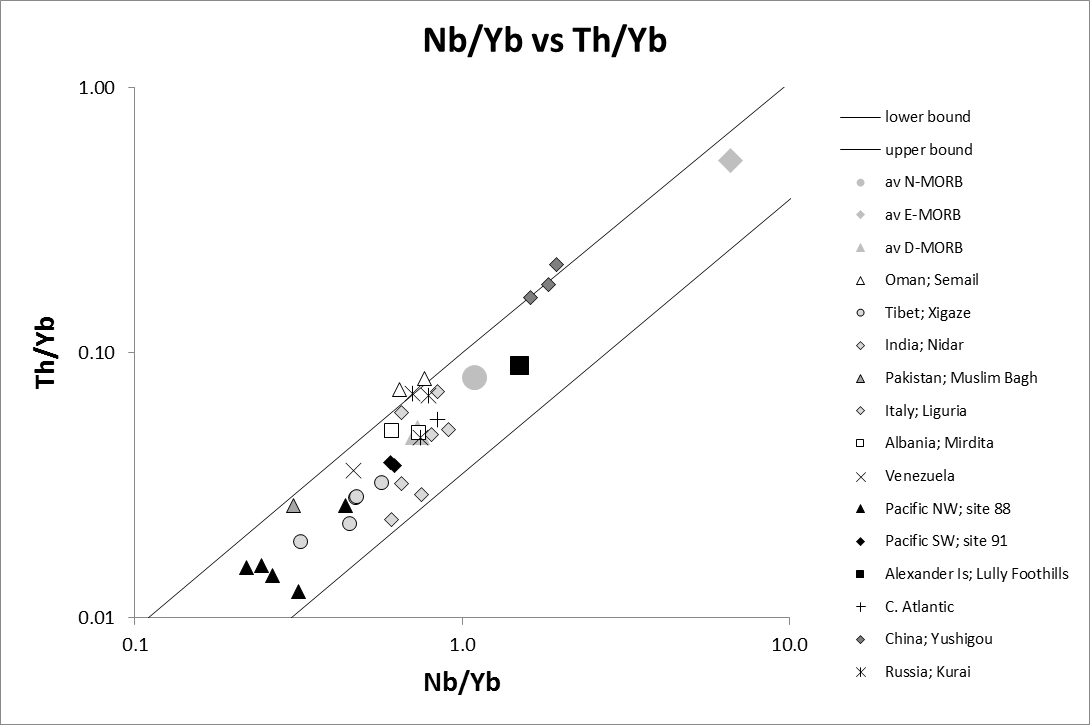


Supplementary Information Fig. S16(b). Nb/Yb versus Th/Yb showing ophiolite samples plotting dominantly within the MORB array. Those samples plotting on or slightly above the upper bound indicate a slight influence of fluid contamination (after ^22^).


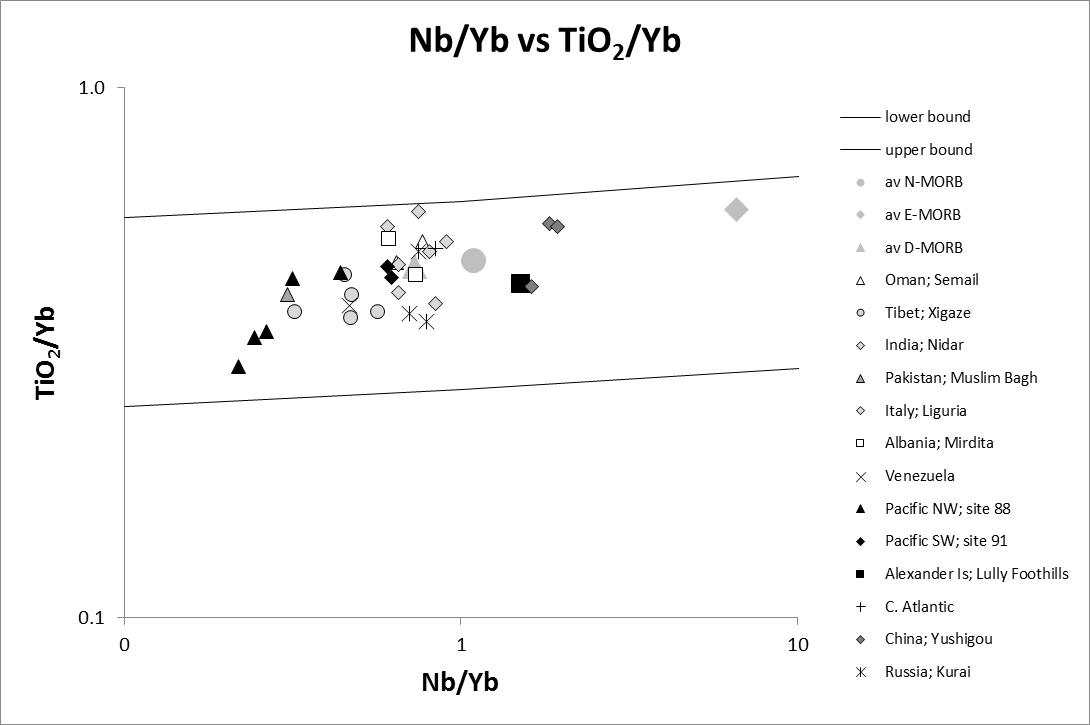


Supplementary Information Fig. S16 (c). Nb/Yb versus TiO_2_/Yb. All the ophiolite and old ocean floor samples plot within the MORB array (after ^22^).


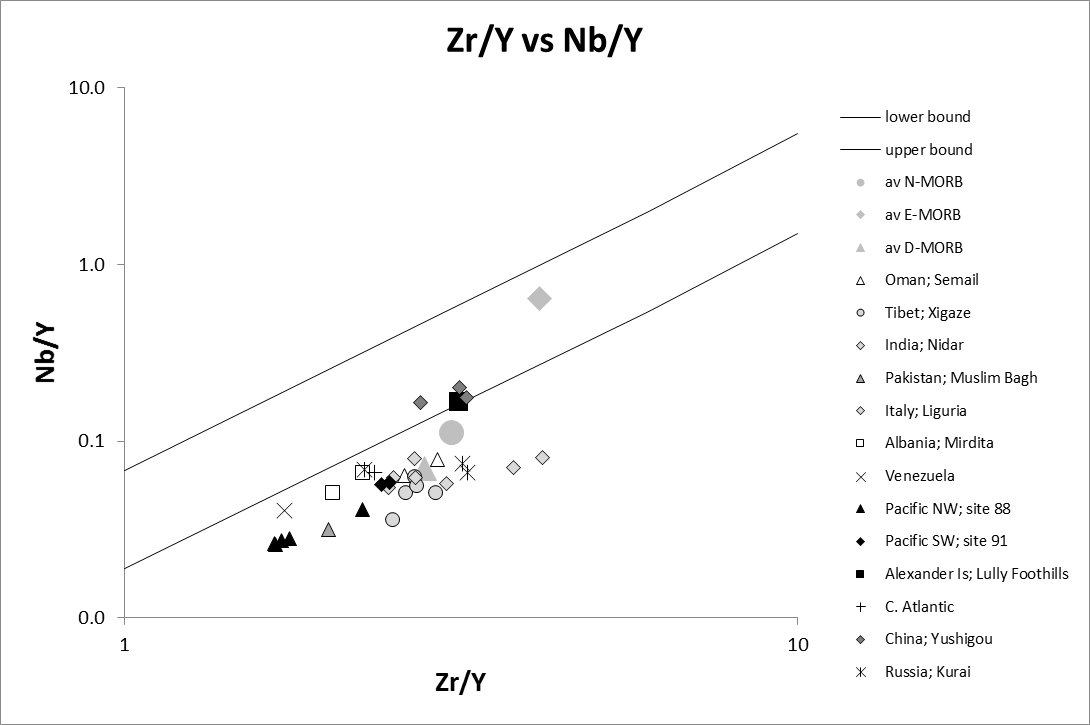


Supplementary Information Fig. S16(d). Zr/Y versus Nb/Y showing most samples plotting below the lower bound indicating similarities with MORB compositions. Four samples plot on or within the array indicating some potential enrichment trend towards E-MORB (after ^23^).


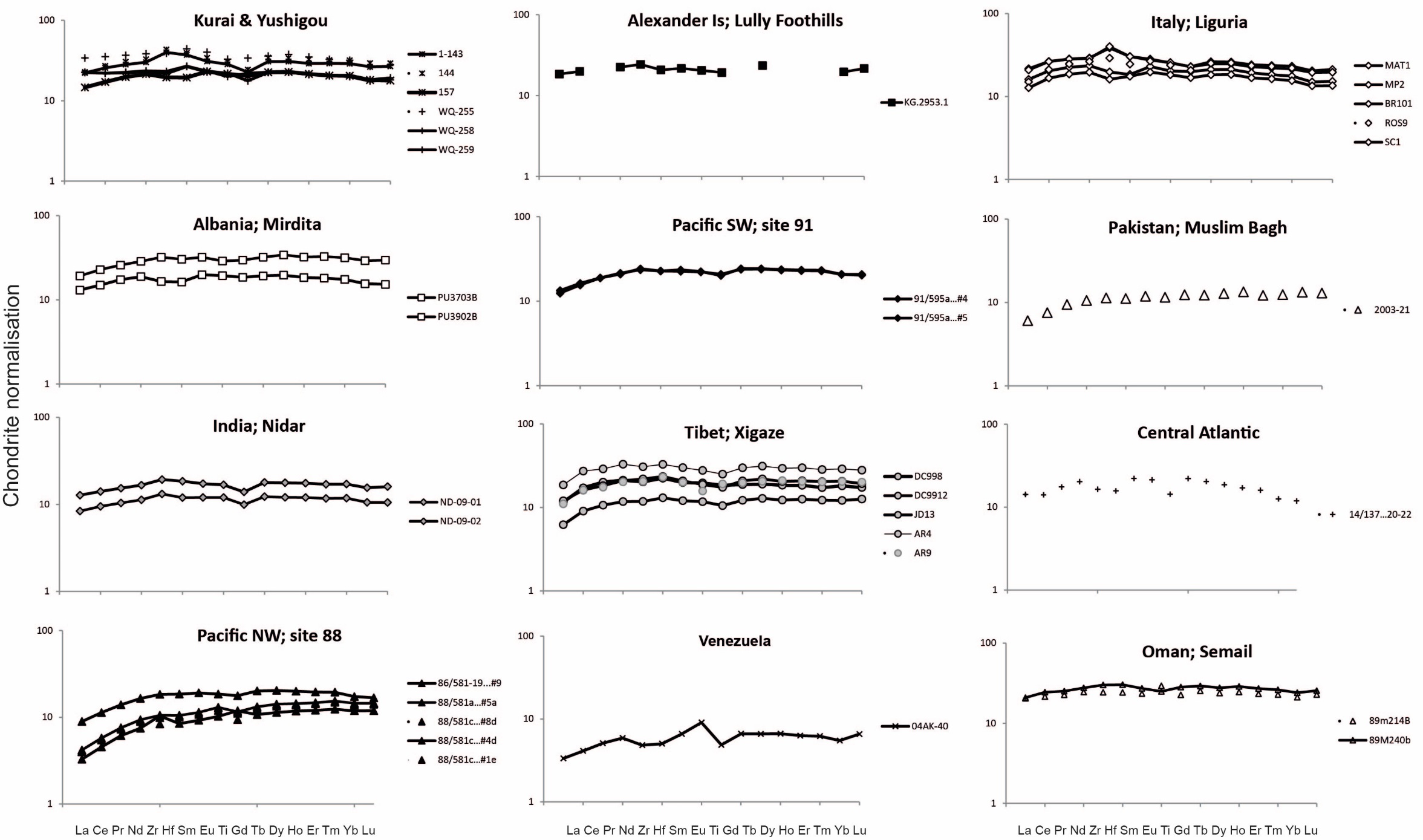


Supplementary Information Fig. S16 (e). Multi-element plots showing relatively flat-lying, MORB-like distribution patterns for each of the ophiolites and old ocean crust included in this study; samples normalised to chondrite^24^.

Supplementary Information Table S4. Data for standard JMC475 measured during Hf isotope batches to assess Hf isotope data quality.

| Session | ^176^Hf/^177^Hf | ± 1σ | n |
| --- | --- | --- | --- |
| Neptune 2010-12-21 | 0.282160 | 0.000007 | 39 |
| Neptune 2011-05-12 | 0.282145 | 0.000007 | 52 |
| Neptune 2011-12-18 | 0.282151 | 0.000010 | 15 |
| Neptune 2012-03-07 | 0.282150 | 0.000005 | 21 |
| Neptune 2013-03-11 | 0.282148 | 0.000004 | 9 |
| Neptune 2013-08-14 | 0.282157 | 0.000008 | 10 |

References

1. Mitrovica, J. X. & Forte, A. M. A new inference of mantle viscosity based upon joint inversion of convection and glacial isostatic adjustment data. *Earth and Planetary Science Letters* **225**, 177-189 (2004).
2. Bello, L., Coltice, N., Tackley, P. J., Müller, R. D. & Cannon, J. Assessing the role of slab rheology in coupled plate-mantle convection models. *Earth and Planetary Science Letters* **430**, 191-201 (2015).
3. Lithgow-Bertelloni, C. & Richards, M.A. The dynamics of Cenozoic and Mesozoic plate motions. *Reviews of Geophysics* **36**, 27-78 (1998).
4. Seton, M. *et al.* Global continental and ocean basin reconstructions since 200 Ma. *Earth-Science Reviews* **113**, 212-270 (2012).
5. Thomson, M. R. A. & Tranter, T. H. Early Jurassic fossils from central Alexander Island and their geological setting. *British Antarctic Survey Bulletin* **70**, 23-39 (1986).
6. Dilek, Y., Furnes, H. & Shallo, M. Geochemistry of the Jurassic Mirdite ophiolite (Albania) and the MORB to SSZ evolution of a marginal basin oceanic crust. *Lithos* **100**, 174-209 (2008).
7. Montanini, A., Tribuzio, R. & Vernia, L. Petrogenesis of basalts and gabbros from an ancient continent-ocean transition (External Liguride ophiolites, Northern Italy). *Lithos* **101**, 453-479 (2008).
8. Rampone, E., Hofmann, A. W. & Raczek, I. Isotopic contrasts within the Internal Liguride ophiolite (n. Italy): the lack of a genetic mantle-crust link. *Earth and Planetary Science Letters* **163**, 175-189 (1998).
9. Sanfilippo, A. & Tribuzio, R. Melt transport and deformation history in a nonvolcanic ophiolitic section, northern Apennines, Italy: implications for crustal accretion at slow spreading settings. *Geochemistry Geophysics Geosystems* **12**, Q0AG04, doi:1029/2010GC003429 (2011).
10. Saunders, A. D. 15. Geochemistry of basalts from Mesozoic Pacific Ocean crust: deep sea drilling project Leg 91. In: Menard, H.W., Natland, J., Jordan, T.H., Orcutt, J.A., *et al*., *Initial Reports of the Deep Sea Drilling Project*, 483-494 (1987).
11. Khan, M., Kerr, A. C. & Mahmood, K. Formation and tectonic evolution of the Cretaceous-Jurassic Muslim Bagh ophiolitic complex, Pakistan: implications for the composite tectonic setting of ophiolites. *Journal of Asian Earth Sciences* **31**, 112-127 (2007).
12. Ahmad, T., Tanaka, T., Sachan, H. K., Asahara, Y., Islam, R. & Khanna, P. P. Geochemical and isotopic constraints on the age and origin of the Nidar ophiolitic complex, Ladakh, India: implications for the Neo-Tethyan subduction along the Indus suture zone. *Tectonophysics* **451**, 206-224 (2008).
13. Niu, X. L., Zhao, Z. D., Mo, X. X., DePaolo, D. J., Zhang, S. Q., Zhu, D. C. & Guo, T. Y. Elemental and Sr-Nd-Pb isotopic geochemistry for basic rocks from Decun-Angren ophiolites in Xigaze area, Tibet: implications for the characteristics of the Tethyan upper mantle domain. *Acta Petrologica Sinica* **22**, 2875-2888 (2006).
14. Burky, D. Neogene coccolith stratigraphy, Mid-Atlantic Ridge, Deep Sea Drilling Project Leg 45, in Melson, W. G., Rabinowitz, P. D. *et al.,* eds., *Init. Rep. Deep Sea Drilling Project* **45**, Washington, D.C., U.S. Government Printing Office, 307-317 (1978).
15. Beccaluva, L., Coltorti, M., Giunta, G., Iturralde Vinent, M., Navarro, E., Siena, F. & Urbani, F. Cross-sections through the ophiolitic units of the southern and northern margins of the Caribbean Plate in Venezuela (northern Cordillera) and Central Cuba. *Ofioliti* **21**, 85–103 (1996).
16. Giunta, G., Beccaluva, L., Coltorti, M., Siena, F. & Vaccaro, C. The southern margin of the Caribbean Plate in Venezuela: tectono-magmatic setting of the ophiolitic units and kinematic evolution. *Lithos* **63***,* 19-40 (2002).
17. Godard, M., Bosch, D. & Einaudi, F. A MORB source for low-Ti magmatism in the Semail ophiolite. *Chemical Geology* **234**, 58-78 (2006).
18. Safonova, I.Y., Simonov, V.A., Buslov, M.M., Ota, T. & Maruyama, S. Neoproterozoic basalts of the Paleo-Asian Ocean (Kurai accretionary zone, Gorny Altai, Russia): geochemistry, petrogenesis, and geodynamics. *Russian Geology and Geophysics* **49**, 254-271 (2008).
19. Shi, R., Yang, J.S. & Wu, C. L. First SHRIMP dating for the formation of the Late Sinian Yushigou ophiolite, North Qilian Mountains. *Acta Geologica Sinica (in Chinese)* **78**, 649-657 (2004).
20. Hou, Q., Zhao, Z., Zhang, H., Zhang, B. & Chen, Y. Indian Ocean MORB-type isotopic signature of Yushigou ophiolite in North Qilian mountains and its implications. *Science in China: Series D Earth Sciences* **49**, 561-572 (2006).
21. Pearce, J. A. & Cann, J. R. Tectonic setting of basic volcanic rocks determined using trace element analyses. *Earth and Planetary Science Letters* **19**, 290-300 (1973).
22. Pearce, J. A. Geochemical fingerprinting of oceanic basalts with applications to ophiolite classification and the search for Archean oceanic crust. Lithos ***100***, 14-48 (2008).
23. Fitton, J. G., Saunders, A.D., Norry, M.J., Hardarson, B.S. & Taylor, R.N. Thermal and chemical structure of the Iceland plume. *Earth and Planetary Science Letters* **153**, 197-208 (1997).
24. Sun, S. -S. & McDonough, W. F. Chemical and isotopic systematics of oceanic basalts: implications for mantle composition and processes. From Saunders, A.D. and M.J. Norry, (eds), 1989, Magmatism in the ocean basins, *Geological Society Special Publication* **42**, 313-345 (1989).
